# Hepatitis B virus-related intrahepatic cholangiocarcinoma originates from hepatocytes

**DOI:** 10.1007/s12072-023-10556-3

**Published:** 2023-06-27

**Authors:** Zimin Song, Shuirong Lin, Xiwen Wu, Xiaoxue Ren, Yifan Wu, Haoxiang Wen, Baifeng Qian, Haozhong Lin, Yihao Huang, Chenfeng Zhao, Nian Wang, Yan Huang, Baogang Peng, Xiaoxing Li, Hong Peng, Shunli Shen

**Affiliations:** 1 Center of Hepato-Pancreato-Biliary Surgery, The First Affiliated Hospital of Sun Yat-Sen University, Guangzhou, 510030 China; 2 Department of Clinical Nutrition, State Key Laboratory of Oncology in South China, Collaborative Innovation Center for Cancer Medicine, Sun Yat-Sen University Cancer Center, Guangzhou, 510060 Guangdong People’s Republic of China; 3 Department of Oncology, The First Affiliated Hospital of Sun Yat-Sen University, Guangzhou, 510030 China; 4 Department of Laboratory Medicine, The First Affiliated Hospital of Sun Yat-Sen University, Guangzhou, 510030 China; 5 Zhongshan School of Medicine, Sun Yat-Sen University, Guangzhou, 510030 China; 6 Institute of Precision Medicine, the First Affiliated Hospital of Sun Yat-Sen University, Guangzhou, 510030 China

**Keywords:** HBV, Intrahepatic cholangiocarcinoma (ICC), Organoids, Hepatocytes

## Abstract

**Background:**

Hepatitis B virus (HBV) infection is one of the most common risk factors for intrahepatic cholangiocarcinoma (ICC). However, there is no direct evidence of a causal relationship between HBV infection and ICC. In this study, we attempted to prove that ICC may originate from hepatocytes through a pathological study involving ICC tissue-derived organoids.

**Method:**

The medical records and tumor tissue samples of 182 patients with ICC after hepatectomy were collected. The medical records of 182 patients with ICC were retrospectively analyzed to explore the prognostic factors. A microarray of 182 cases of ICC tumor tissue and 6 cases of normal liver tissue was made, and HBsAg was stained by immunohistochemistry (IHC) to explore the factors closely related to HBV infection. Fresh ICC tissues and corresponding adjacent tissues were collected to make paraffin sections and organoids. Immunofluorescence (IF) staining of factors including HBsAg, CK19, CK7, Hep-Par1 and Albumin (ALB) was performed on both fresh tissues and organoids. In addition, we collected adjacent nontumor tissues of 6 patients with HBV (+) ICC, from which biliary duct tissue and normal liver tissue were isolated and RNA was extracted respectively for quantitative PCR assay. In addition, the expression of HBV-DNA in organoid culture medium was detected by quantitative PCR and PCR electrophoresis.

**Results:**

A total of 74 of 182 ICC patients were HBsAg positive (40.66%, 74/182). The disease-free survival (DFS) rate of HBsAg (+) ICC patients was significantly lower than that of HBsAg (−) ICC patients (*p* = 0.0137). IF and IHC showed that HBsAg staining was only visible in HBV (+) ICC fresh tissues and organoids, HBsAg expression was negative in bile duct cells in the portal area. Quantitative PCR assay has shown that the expression of HBs antigen and HBx in normal hepatocytes were significantly higher than that in bile duct epithelial cells. Combined with the IF and IHC staining, it was confirmed that HBV does not infect normal bile duct epithelial cells. In addition, IF also showed that the staining of bile duct markers CK19 and CK7 were only visible in ICC fresh tissue and organoids, and the staining of hepatocyte markers Hep-Par1 and ALB was only visible in normal liver tissue fresh tissue. Real-time PCR and WB had the same results. High levels of HBV-DNA were detected in the culture medium of HBV (+) organoids but not in the culture medium of HBV (−) organoids.

**Conclusion:**

HBV-related ICC might be derived from hepatocytes. HBV (+) ICC patients had shorter DFS than HBV (−) ICC patients.

**Supplementary Information:**

The online version contains supplementary material available at 10.1007/s12072-023-10556-3.

## Introduction

Intrahepatic cholangiocarcinoma (ICC) is the second most common intrahepatic malignancy [1, 2]. It has an increasing morbidity and mortality rates worldwide [3, 4]. Surgical resection is the recommended first-line treatment for early-stage ICC[5], while most patients have lost the chance of surgery at the time of diagnosis. Systemic chemotherapy is preferred for late-stage ICC, but its efficacy is limited [6].Therefore, a deeper understanding of the pathogenesis of ICC is becoming increasingly important, which provides the possibility for the early diagnosis and treatment of ICC.

Hepatitis B virus (HBV) is one of the risk factors for ICC [7–9].Our previous study suggested that serum HBsAg staining was positive in 27.7% of ICC patients [10, 11]. In Shen’s report, up to 77.2% of ICC cases (564/731) were complicated with HBV infection[12]. In addition, 69% of patients with mixed liver cancer had HBV infection [13]. Furthermore, as many as 70.4% of the tumor tissues from HBV-positive ICC patients expressed HBx protein [14]. HBV is characterized by obvious liver tropism and only invades hepatocytes [15, 16]. Therefore, all these studies indicate that HBV-associated ICC likely originates from hepatocytes.

There has been much controversy over whether hepatocytes can be malignantly transformed into ICC [17]. It is generally accepted that ICC originates from bile duct epithelial cells [18, 19]. However, with the development of genetically engineered mouse models and lineage tracing technology, strong evidence has shown that ICC can be derived from hepatocytes. Sekiya et al. crossed Alb-Cre-jER^T2^ (with labelled hepatocytes) and CK19-Cre ER^T2^ (with labelled cholangiocytes) mice with mice expressing R26R^lacz/Lacz^ or R26R^YFP/YFP^. Then, a thioacetamide (TAA)-induced liver injury model was constructed. By detecting the tracer markers lacZ or YFP, they found that hepatocytes around the portal vein were labelled and transdifferentiated into CK19 ( +) bile duct cells after 14 weeks, which eventually developed into ICC after 30 weeks, while CK19-labelled bile duct cells did not develop into ICC [17]. Researchers activated Notch1 and AKT genes in liver cells in mice using hydrodynamic injection transfection (HDT), and after 1.5 weeks, malignant transformation of hepatocytes was observed. After 4.5 weeks, tumor nodules with typical ICC characteristics developed. These nodules mainly appeared in the central hepatic lobule area but not in the portal area where bile duct cells were clustered [20]. Similarly, Wang et al. transfected AKT and YAP genes into mouse hepatocytes by the HDT method and successfully induced ICC [21]. Seehawer et al. also found that the necroptosis microenvironment can induce malignant transformation of hepatocytes to form ICC [22].

In this study, we attempted to prove that ICC may originate from hepatocytes through a pathological aspect, providing information on the pathogenesis of ICC.

## Materials and methods

### Patients and ICC tissue specimens

Patients with prior malignant tumor history, co-infection with HCV or/and HDV, autoimmune liver disease, alcohol-related liver disease and other liver diseases and incomplete data were excluded from this study. A total of 182 patients with first diagnosed ICC who underwent hepatectomy at the First Affiliated Hospital of Sun Yat-sen University from April 2004 to September 2015 were included in this study. ICC diagnosis was confirmed by pathology in all patients. ICC pathological diagnosis criteria followed WHO Pathological Classification of Liver and intrahepatic Bile Duct Tumors (2019 edition) [23, 24]. Patients ranged in age from 24 to 82 years. There were 98 males and 84 females. All patients had complete clinical and laboratory data. None of the patients received any type of antitumor therapy before surgery. The diagnosis and treatment of ICC mainly comply to the Chinese Expert Consensus on the Diagnosis and Treatment of Intrahepatic Cholangiocarcinoma (2022 Edition) [25]. Radical resection of the tumor (R0 resection) and preservation of sufficient functional residual liver volume are the principles of ICC surgical resection. For stage IB and stage II ICC without vascular invasion, anatomic hepatectomy is recommended after rigorous evaluation. For ICC with large tumor volume, multiple lesions, and complicating large vessel invasion, neoadjuvant, conversion therapy or extended hepatectomy will be performed after multi-disciplinary treatment (MDT) discussion, so as to obtain the opportunity of radical resection. Gemcitabine combined with cisplatin is the first-line treatment for advanced ICC.

ICC patients who underwent surgery were followed up once every 3 months for 2 years after surgery, once every 6 months for 2 to 5 years after surgery, and once a year after 5 years. Each follow-up examination included: (1) general physical examination; (2) Imaging examination: upper abdominal enhanced CT or MRI with intermittent lung CT scan. PET-CT will be arranged when necessary. (3) Laboratory examination: routine blood examination, blood biochemistry, CA19‑9, CEA and other tumor markers. (4) In the case of HBV (+), hepatitis B viral load, hepatitis B-related antibodies and antigens, and liver function need to be tested. These patients are routinely treated with antiviral therapy.

When collecting fresh tissue specimens, the excised gross specimen should first be observed and photographed to confirm the location and scope of the tumor. Generally, the specimen should be retrieved less than 30 min after removal from the abdomen. Tumor and adjacent tissues were treated separately, and they were cut into several tissue blocks with a diameter of about 0.5 cm, which were put into sterile freezer-storage tubes, quickly put into liquid nitrogen, and then transferred into liquid nitrogen tanks for long-term preservation. In addition, some tumor and paracancer tissues should be collected and transported and preserved in 4% paraformaldehyde for later tissue microarray and paraffin section construction. In the process of tissue specimen collection, patient information should be marked and recorded. We made a tissue microarray as previously reported [26] and performed IHC staining for HBsAg protein. Then, the results of IHC staining were scored, and the patients were divided into the HBsAg positive expression group [HBsAg (+)] and the HBsAg negative expression group [HBsAg (−)] according to the score.

In addition, ICC tissues and corresponding adjacent tissues were collected from 3 HBsAg (+) patients and 3 HBsAg (−) patients, respectively. Then, the collected tissues were used to make paraffin sections and cultured organoids. Next, we performed IF assays on paraffin sections and organoids for HBsAg protein, CK19 protein, CK7 protein, ALB protein and Hep-Par1 protein respectively. Tissues were selected from the Center of Hepato-Pancreato-Biliary Surgery, the First Affiliated Hospital of Sun Yat-sen University. The informed consent has been obtained from all patients. This study was approved by the Research Medical Ethics Committee of the First Affiliated Hospital of Sun Yat-sen University (Ethics number: [2022]003) and followed the ethical guidelines of the Declaration of Helsinki.

### Immunohistochemistry (IHC)

The paraffin-embedded tissue microarrays were first placed in an oven at 65 °C for 2 h. After baking, the tissue microarray was quickly dewaxed in xylene 3 times for 15 min each time. The tissue microarrays were then sequentially rehydrated in graded ethanol. Endogenous peroxidase activity was blocked with 3% catalase for 10 min. Tissue microarrays were soaked in citrate buffer (pH 6.0) and heated in a microwave oven at 100 °C for 15 min to repair antigens. Between the above operations, tissue microarrays were rinsed with phosphate buffered saline (PBS) three times for 5 min each time. After blocking treatment with 10% goat serum for 30 min, the microarrays were incubated with monoclonal anti-HBsAg antibody (1:50 dilution; Novus Biologicals, Briarwood Avenue, Centennial, CO 80112, USA) at 4 °C overnight. They were then incubated with secondary antibody (GTVision™ III Detection System/Mo&Rb) for 30 min at room temperature and colour-developed with 3,3'-diaminobenzidine hydrochloride (GTVision™ III Detection System/Mo&Rb). Finally, the nuclei were stained with hematoxylin, and the slices were sealed with resin.

We randomly selected 5 fields from each patient’s slide. For each field, the proportion of positively stained cells and the intensity of staining should be assessed, and they should be combined for scoring. A score of 0 was defined as negative expression, 1–4 was defined as weak positive, 5–8 as moderate positive, and 9–12 as strong positive. Finally, the average score of the five visual fields was obtained [27]. Scoring was performed independently by two investigators who were unaware of specific information about tissue microarrays.

### Preparation of organoids from liver and ICC tumor tissues

**Fig. 1:**
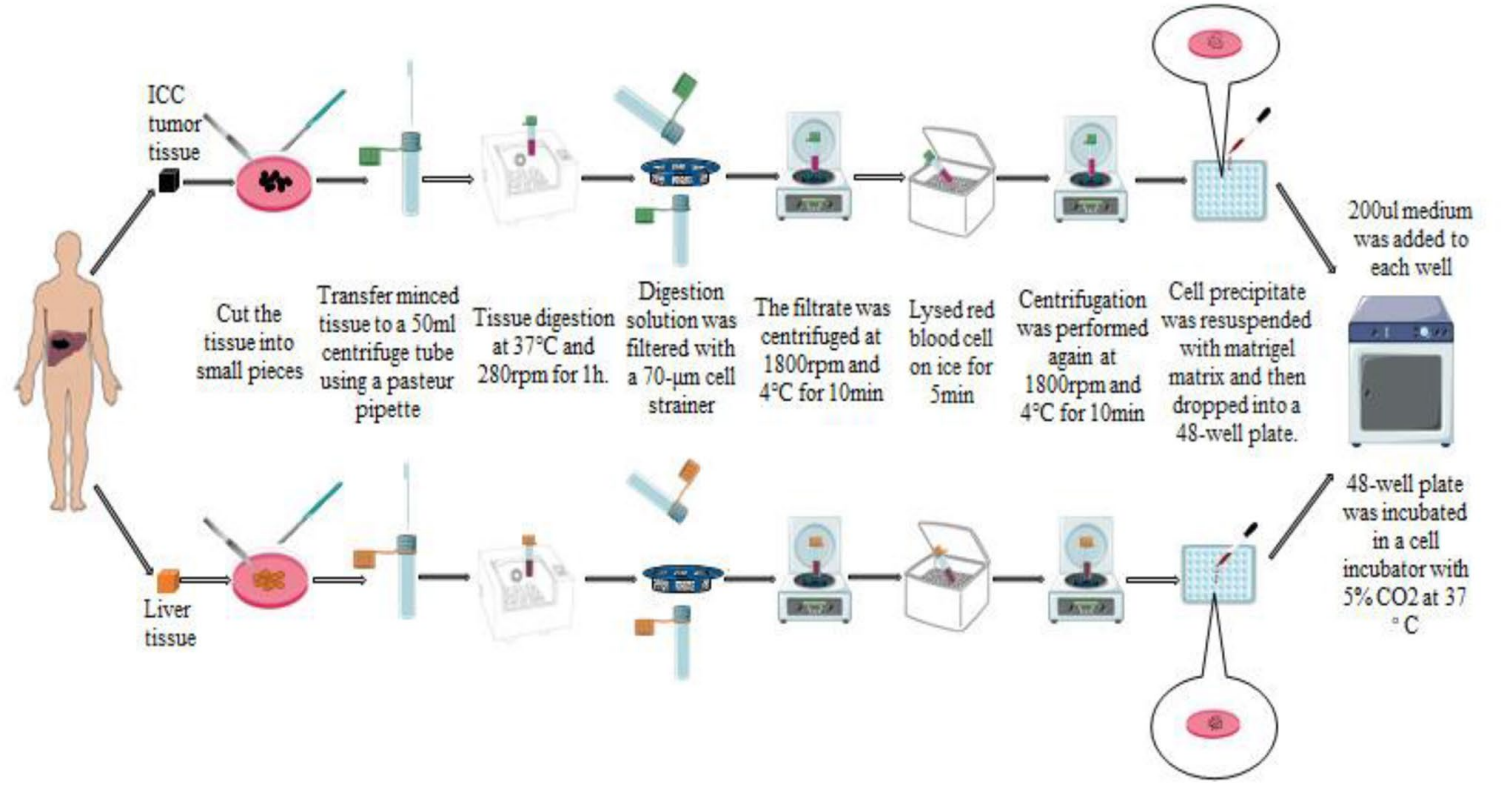
The process of organoid isolation and culture. We referred to the organoid preparation process reported by Hans Clevers et al. [28, 29]

ICC tumor specimens and corresponding adjacent liver tissue should be collected as soon as possible after surgical resection. The principle of sterility should be strictly observed in the process of collection. The collected specimens were placed in basal medium (Advanced DMEM/F-12 (Life Technologies, cat. no. 12634–010) + 1% penicillin/streptomycin (Life Technologies, cat. no. 15140–122) + 1% GlutaMAX (100 × ; Life Technologies, cat. no. 35050–068) + HEPES 10 mM (Life Technologies, cat. no. 15630–056)), stored and transported on ice. Specimens were processed within 30 min after collection. The specimens were washed three times with wash medium (DMEM (Life Technologies, cat. no. 31966–021) + 1% FBS (Life Technologies, cat. no. 26010066) + 1% penicillin/streptomycin.) and then transferred to 10 cm sterile petri dishes. Two to three millilitres of digestive solution (10 ml F12 + 100 µl collagenase D (Roche, cat. no. 1108866001) + 20 µl Primcin™ (InvivoGen, cat. no. ant-pm-1)) were added to each dish to submerge the tissue block. The tissue block was secured with forceps and then cut into small pieces with a surgical blade. The minced tissue was then transferred to a 50 ml centrifuge tube using a 7 ml plastic Pasteur pipette (VWR, cat. no. 612–1681), and the total amount of digestive fluid was 10 ml. Next, the centrifuge tube was placed into a shaker for tissue digestion at 37 °C and 280 rpm for 1 h. When the digestion solution contained 80–100% single cells, the digestion solution was immediately filtered with a 70-μm cell strainer (Falcon, cat. no. 352350), and the filtrate was collected into a new 50 ml centrifuge tube and then DPBS (GIBCO, cat. no. C14190500BT) was added to rinse the filter. The filtrate was centrifuged at 1800 rpm and 4 °C for 10 min. The supernatant was removed, 6 ml of red cell lysate was added to each tube, and the tissue cells were resuspended and placed on ice for 5 min. Centrifugation was performed again at 1800 rpm and 4 °C for 10 min. The supernatant was removed, and as much of the remaining fluid as possible was aspirated. The cell precipitate was resuspended in Matrigel matrix (BD, cat. no. 356231) [28, 29], mixed well, and then the resuspended liquid was dropped into a 48-well plate, adding approximately 30 ml to each well. The specific number of wells and the amount of Matrigel were determined according to the amount of cell precipitation. Then, the 48-well plates were placed upside down in an incubator at 37 °C for 1 h, and 200 µl medium was added to each well. ICC tissues-derived organoid medium composition: basal medium + human liver expansion medium (B27 supplement (without vitamin A) (50 × ; Life Technologies, cat. no. 12587-010), N2 supplement (100 × ; Life Technologies, cat. no. 17502-048), 1 mM *N*-acetylcysteine (Sigma-Aldrich, cat. no. A0737-5MG), 10% (vol/vol) Rspo1-conditioned medium[25], 10 mM nicotinamide (Sigma-Aldrich, cat. no. N0636), 10 nM recombinant human [Leu15]-gastrin I (Sigma-Aldrich, cat. no. G9145), 50 ng/ml recombinant human EGF (Peprotech, cat. no. AF-100-15), 100 ng/ml recombinant human FGF10 (Peprotech, cat. no. 100-26), 25 ng/ml recombinant human HGF (Peprotech, cat. no. 100-39), 10 μM Forskolin (Tocris Bioscience, cat. no. 2939) and 5 μM A83-01 (Tocris Bioscience, cat. no. 2939)). Liver tissues-derived organoid medium composition: 30% wnt3a-conditioned medium [25] + basal medium + human liver expansion medium. Add a circle of DPBS around it. Finally, the 48-well plate was incubated in a cell incubator with 5% CO2 at 37 °C. The process of organoid isolation and culture is shown in Fig. 1.

### Immunofluorescence (IF)

Paraffin sections were prepared after the IF test of tissue paraffin sections. The collected ICC and corresponding paracancerous tissues were fixed in 4% paraformaldehyde (Biosharp, cat. no. BL539A) and embedded in paraffin. Paraffin sections (5 μm) were continuously cut parallel to the maximum section of the tissue. Subsequent baking, dewaxing, antigen repair, inhibition of endogenous peroxidase activity, and blocking were the same as for IHC. The diluted primary antibody (anti-HBsAg, anti-CK19 (1:50 dilution; Signalway Antibody LLC, 6305 Ivy Lane, Suite 370, Greenbelt, Maryland, USA)) was dropped on the tissue and incubated overnight at 4 °C. The temperature was restored at room temperature for 1 h. Tissue sections were washed with PBST (Life Technologies, cat. no 14190-094) + 1% TritonTM X-100 (Sigma‒Aldrich, cat. no 9036-19-5)) 3 times for 10 min each time. The secondary antibody (anti-rabbit IgG (H + L) F (ab')2 fragment (Alexa Fluor 647 Conjugate) (1:500 dilution; Cell Signaling Technology (CST), Danvers, Massachusetts, USA), anti-mouse IgG (H + L), F (ab')2 fragment (Alexa Fluor^®^ 488 Conjugate) (1:500 dilution; CST)) was incubated for 1 h. The cells were rinsed with PBS 3 times for 15 min each time. Paraffin sections were incubated with 4',6-diamidino-2-phenylindole dihydrochloride (DAPI) (CST, Danvers, Massachusetts, USA) for 8 min to stain nuclei. The cells were rinsed with PBS as before. Finally, the paraffin sections were sealed with anti-quench sealing agent. Exposure was performed with an inverted fluorescence microscope. Paraffin sections were observed under an inverted fluorescence microscope and analyzed using DMI 4000B analysis software.

Organoid IF sample preparation and experiments required gentle movements. Before fixing the organoids with 4% paraformaldehyde, the organoids were re-embedded in confocal dishes with Matrigel matrix. After absorption of paraformaldehyde, organoids were washed with PBS 3 times for 5 min each time. Then, 1 ml 0.3% Triton was added to the dish and placed on a shaker for 20 min at approximately 50–60 rpm. After the Triton was aspirated, the organoids were washed three times with PBS again. Then, 20% goat serum was added to the dish and incubated for 1 h at room temperature. Subsequent primary antibody incubation, secondary antibody incubation, and nuclear staining were performed in the same way as the IF assay of paraffin sections. Organoids were observed under a fluorescence microscope and analyzed using ZEN (2.3 SP1) analysis software.

### Tissue protein extraction

First, the cell lysate was prepared (1 ml working solution = 1 ml RIPA (EpiZyme, cat. no. PC101) + 10ul Protease/Phosphatase Inhibitor Cocktail (100 × ; CST, cat. no. 5872S)) and placed on ice for later use. Chop the fresh tissues into fragments with a diameter of about 1–2 mm, grind them into powder, add 500ul cell lysis solution, and thoroughly mix. Leave it on ice for 30 min to allow the cells to break down sufficiently. Centrifuge in high speed centrifuge, centrifuge conditions: 4 °C, 14000 rpm, 15 min, supernatant was collected, the total concentration of extracted protein was detected by BCA protein quantitative kit (EpiZyme, cat. no. ZJ101), and stored at -80℃ for later use.

### Western blot (WB)

An equal amount of total protein was run on 10% SDS-PAGE (EpiZyme, cat. no. PG112), transferred to PVDF membranes (Merck millipore, cat. no. IPVH00010) (380 mA for 2 h), and probed with primary antibodies. The primary antibodies include anti-HBsAg (1:1000, 27 kDa, Novus Biologicals), anti-HBx (1:1000, 17 kDa, Abcam), anti-CK7 (1:1000, 51 kDa, Signalway antibody (SAB)), anti-CK19 (1:1000, 40 kDa, Signalway antibody (SAB)), anti-Hep-Par1 (1:1000, 165 kDa, Proteintech Group), anti-ALB (1:1000, 66 kDa, Proteintech Group), anti-α-Tubulin (1:1000, 55 kDa, Proteintech Group), anti-GAPDH (1:1000, 36 kDa, CST). The target protein bands were captured by binding of the secondary antibodies linked with peroxidase (1:1000, Anti-Rabbit IgG (H + L), CST) (1:1000, anti-mouse IgG (H + L), CST) to the primary antibodies.

### Tissue RNA extraction and RNA reverse transcription to form cDNA

Total RNAs were extracted using the RN001 RNA Quick Purification kit (ESscience, Shanghai, China) following the instructions. RNA quantity and purity were estimated using a NanoDrop 2000 Spectrophotometer (NanoDrop 2000, Thermo Scientific, American). cDNA was synthesised using PrimeScriptTM RT Master Mix (Perfect Real Time) (Code No. RR036A,TaKaRa, Shiga, Japan).

### Quantitative real-time PCR (qPCR)

A two-step RT-qPCR was performed using SYBR Premix Ex TaqTM II (TaKaRa, Japan) and CFX Connect System (Bio-Rad, American). The amplification protocol was as follows: 95 °C for 30 s, followed by 40 cycles of 95 °C for 5 s and 60 °C for 30 s, lastly followed by 95 °C for 10 s and a melt curve of 65 °C for 5 s and 95 °C for 5 s. Primers used for RT-qPCR are listed in Supplementary Table 1. Glyceraldehyde-3-phosphate dehydrogenase (GAPDH) was used as a reference. The relative expression level of each gene was normalized to tissue that acts as a negative control and calculated using the formula 2 (− ΔΔCt).

### PCR electrophoresis

First, 1.5% agarose gel was prepared to isolate DNA fragments. Add the agarose powder (0.7 g) (Biowest Agarose, cat. no. BY-R0100) and 70 mL 1xTAE electrophoresis buffer (Servicebio, cat. no. G3001) into the conical flask, shake well, and microwave the mixture to boiling. Repeat the heating for 3 times, and the liquid is clear and clear. When the liquid is cooled to 40–50 °C, add 5 μl CelRed dye (Accurate Biology, cat. no. AG1198) into it, shake it gently, pour the agarose liquid gel into the glue plate with a comb inserted, and let it cool and solidify naturally. Secondly, prepare the electrophoresis system (10 μl): (1) PCR sample:9 μl,10 × Loading Buffer: 1 μl (Accurate Biology, cat. no. AG11903); (2) DL 2000 DNA Marker 10 μl (Accurate Biology, cat. no. AG11904). Third, the prepared electrophoresis system was added into the sample hole of agarose gel. Electrophoresis conditions:160 V, 40 min. Finally, the agarose gel was transferred to the gel imaging system for DNA band imaging and the imaging results were preserved.

### Detection of HBV-DNA in organoid culture medium

Supernatants were collected during the change in organoid culture medium. Hepatitis B viral load in culture medium was tested according to the instructions of the hepatitis B virus nucleic acid assay kit (Da’an Gene, Cat. No. 03.02.01.10035). In addition, the supernatants of 3 HBV ( +) ICC derived organoids and 3 HBV (−) ICC derived organoids were collected for PCR electrophoresis to verify the presence of HBV-DNA.

### Statistical analysis

SPSS 23.0 software (IBM, International Business Machines Corp, Chicago, USA) was used for statistical analysis in this study. Measurement data are expressed as the mean ± SE and were compared by Student's *t* test or one-way ANOVA. Measurement data with a nonnormal distribution were compared by a Mann‒Whitney *U* test. The *χ*^2^ test or Fisher's exact test was used to compare categorical data, and Pearson's correlation analysis was used to explore the correlation between variables. The Kaplan‒Meier method was used to draw the survival curves of patients, and the log-rank test was used to compare the survival differences between groups. Variables with statistical significance in univariate analysis (*p* < 0.05) were substituted into the Cox proportional hazards model to explore independent prognostic factors. *p* < 0.05 (two-sided) was considered statistically significant.

## Results

### HBV infection in ICC

**Fig. 2:**
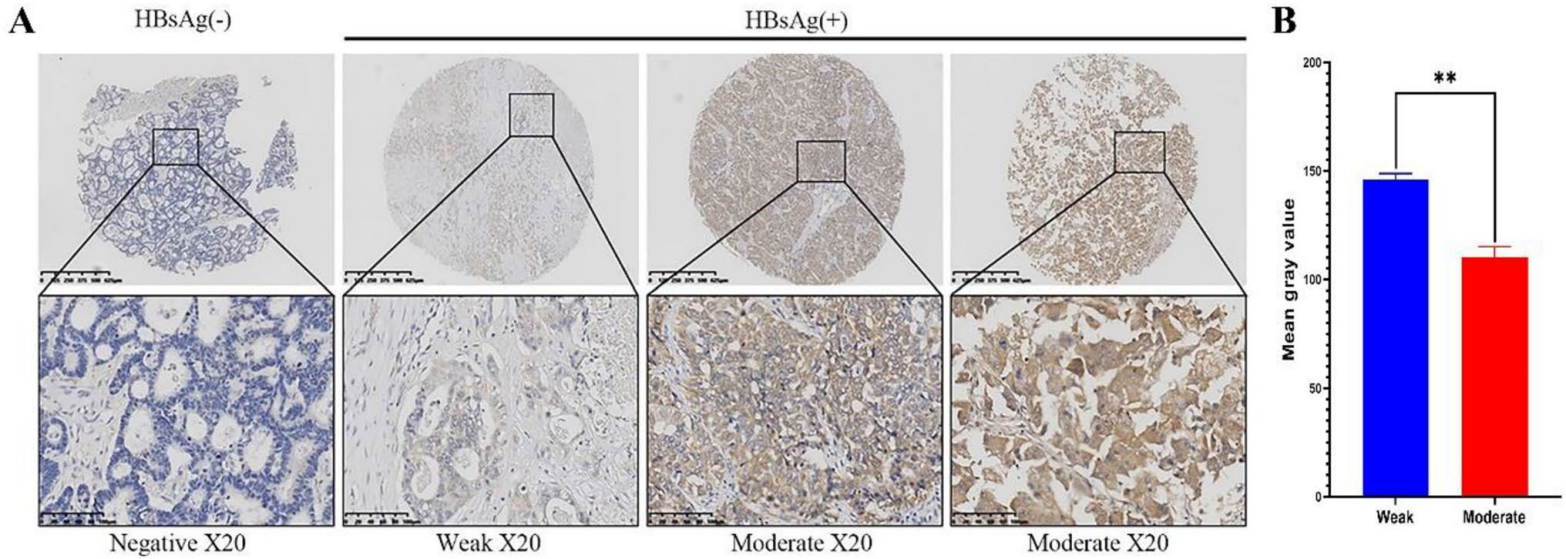
A. The representative staining results were shown. B. The IHC results were quantized by imageJ

First, we assessed the expression level of HBsAg in 182 ICC samples on a tissue microarray by IHC staining. Seventy-four of 182 ICC cases were HBsAg positive (40.66%), and the remaining 108 cases were HBsAg negative (59.34%). The representative staining results are shown in Fig. 2A, the IHC results were quantized by imageJ (Fig. 2B).

The positive rate of HBsAg staining was approximately 40.66%, according to the statistical results of serological detection, our research group previously concluded that the positive rate of HBsAg staining was 27.7%, which was lower than that in this study. It was possible that some patients had low serological concentration of HBsAg, which could not be detected. Then, HBsAg expression in tumor lesions was explored by IHC staining. IHC staining is more sensitive than serological detection. According to the results of IHC staining, 182 ICC patients were divided into HBsAg ( +) positive group (*n* = 74) and HBsAg (−) negative group (*n* = 108). Next, we explored the differences in clinical baseline data and pathological characteristics between HBsAg (+) and HBsAg (−) groups (Table 1**)**. The results showed that there were no significant differences in age, sex, liver cirrhosis and other clinical baseline data between the two groups (*p* > 0.05, Table 1). However, there was a significant difference in the number of neutrophils between the two groups (*p* = 0.005). The level of neutrophils in the HBsAg (+) group was significantly higher than that in HBsAg (−) group, and the level of neutrophils in the HBsAg (+) group was approximately 5.586 ± 2.167 × 10^9/L. The level of neutrophil in the HBsAg (−) group was 4.680 ± 1.843 × 10^9/L. There was no significant difference in pathological characteristics between the two groups (*p* > 0.05).

**Table 1:** Results of differences in clinical baseline data and pathological features between the HBsAg+ and HBsAg(−) groups

Variables	No	HBsAg		*χ*^2^/t	*p* value
		(−) (*n* = 108)	(+) (*n* = 74)		
Gender					
Female	84	54 (50.0)	30 (40.5)	1.581	0.209
Male	98	54 (50.0)	44 (59.5)		
Age (year)		55.954 ± 9.513	57.851 ± 12.332	1.116	0.267
Blood loss (ml)		640.880 ± 819.981	626.351 ± 650.249	− 0.350	0.726
Cirrhosis					
No	140	87 (80.6)	53 (71.6)	1.974	0.160
Yes	42	21 (19.4)	21 (28.4)		
Tumor number					
Single	125	75 (69.4)	50 (67.6)	0.072	0.789
Multiple	57	33 (30.6)	24 (32.4)		
Tumor size (cm)		6.232 ± 2.644	7.142 ± 3.513	− 1.596	0.110
Differentiation^a^					
W + M	128	79 (73.1)	49 (66.2)	1.011	0.315
P	54	29 (26.9)	25 (33.8)		
Tumor stage					
I + II	119	70 (64.8)	49 (66.2)	0.038	0.845
III + IV	63	38 (35.2)	25 (33.8)		
Resection Margin					
R0	75	48 (44.4)	27 (36.5)	1.148	0.284
R1	107	60 (55.6)	47 (63.5)		
TNM^b)^					
I + II	77	43 (39.8)	34 (45.9)	0.676	0.411
III + IV + V	105	65 (60.2)	40 (54.1)		
CEA (ug/l)		51.776 ± 314.546	35.165 ± 223.068	− 1.447	0.148
CA199 (U/ml)		2417.839 ± 4188.052	1394.021 ± 3028.448	− 1.006	0.314
AFP (ug/l)					
Positive		7 (6.5)	10 (13.5)	2.564	0.109
Negative		101 (93.5)	64 (86.5)		
PT (s)		12.238 ± 1.301	12.386 ± 1.100	− 1.220	0.223
PLT (× 10^9/L)		247.287 ± 83.485	261.338 ± 95.225	− 0.586	0.558
Neutrophil (× 10^9/L)		4.680 ± 1.843	5.586 ± 2.167	− 2.813	**0.005**^c)^
Lymphocyte (× 10^9/L)		1.845 ± 0.670	1.787 ± 0.561	− 0.500	0.617
TBIL (umol/L)		33.259 ± 83.346	27.288 ± 51.681	− 0.652	0.515
DBIL (umol/L)		19.776 ± 61.124	15.493 ± 40.087	− 0.153	0.878
ALB (g/L)		39.994 ± 4.174	38.651 ± 6.384	− 1.650	0.099
ALT (U/L)		45.528 ± 54.123	42.581 ± 57.047	− 0.586	0.558
AST (U/L)		44.620 ± 46.849	37.162 ± 32.002	− 0.847	0.397

Bold marks indicated that the results were statistically significant (*P *< 0.05)

*W + M* well + moderately differentiated, *P* poorly differentiated, *AST* aspartate aminotransferase, *ALT* alanine transaminase, *CEA* carcinoembryonic antigen, *CA19-9* carbohydrate antigen 19–9, *AFP* alpha-fetoprotein

^a^Based on the World Health Organization (WHO) classification of tumors of the digestive system 2010

^b^Based on seventh edition cancer staging manual of the American Joint Committee on Cancer

^c^*p* < 0.05

### HBV infection is associated with disease progression and poor prognosis in ICC patients

**Fig. 3:**
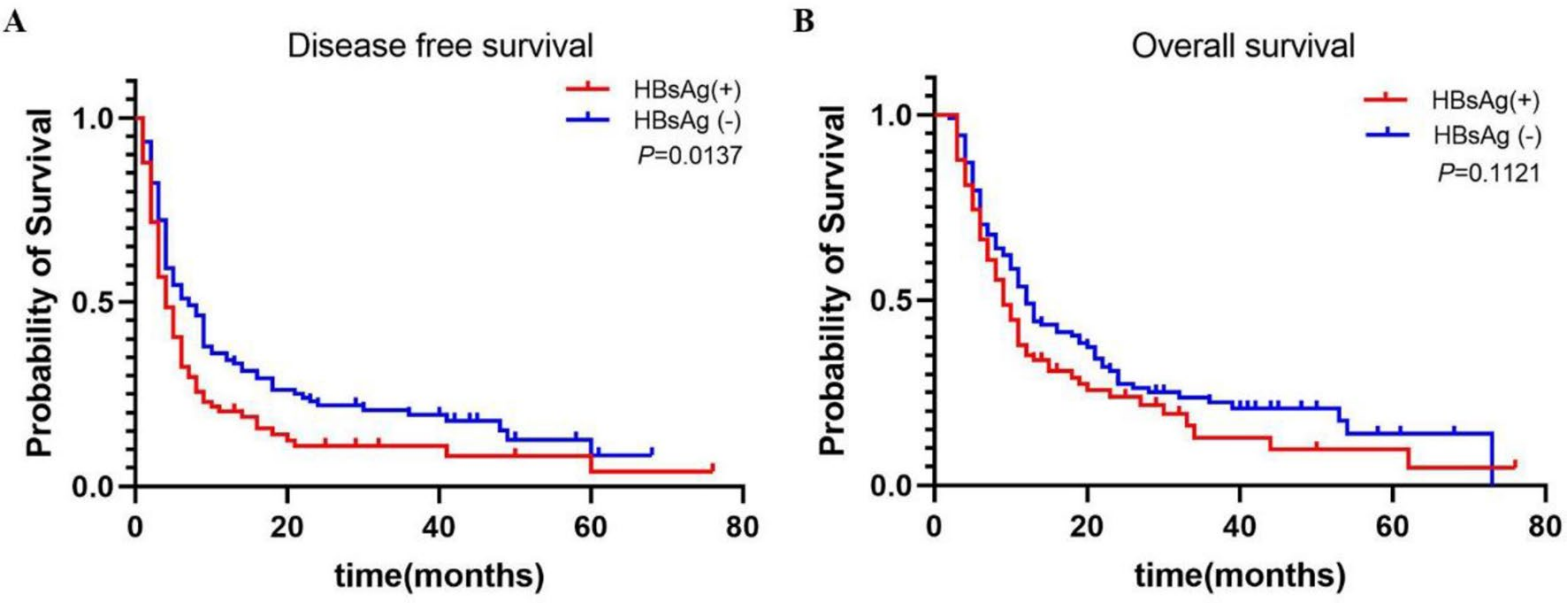
The DFS and OS of 182 ICC patients are shown in **a** and **b**, respectively

Survival analysis showed that disease-free survival (DFS) of HBsAg ( +) was significantly lower than that of HBsAg (−) (*p* = 0.0137, Fig. 3A).The overall survival (OS) of HBsAg (+) patients tended to be lower than that of HBsAg (−) patients, although there was no significant difference (*p* = 0.1121, Fig. 3B). Median DFS was significantly shorter in the HBsAg (+) group than that in the HBsAg (−) group (4 months vs. 7 months). Median OS was also lower in the HBsAg (+) group than in the HBsAg (−) group (9 months vs. 12 months).

A Cox regression proportional hazards model was used for multivariate analysis of prognosis. The results showed that liver cirrhosis (*p* = 0.006), tumor number (*p* = 0.014), tumor size (*p* = 0.045), TNM stage (*p* < 0.001), CEA (*p* = 0.011) and HBsAg expression (*p* = 0.011) were independent prognostic predictors of DFS in ICC patients (Table 2). Tumor number (*p* = 0.035), tumor size (*p* = 0.039), tumor differentiation (*p* < 0.001) and TNM stage (*p* < 0.001) were independent prognostic factors for OS (Table 2). These results indicate that HBV-infected ICC patients have rapid disease progression and a poorer prognosis than HBV-negative patients.

**Table 2:** Results of Cox regression proportional risk model for multivariate prognostic analysis

Variables	Overall survival (OS)			Disease-free survival (DFS)		
	Univariate *p* value	Multivariate		Univariate *p* value	Multivariate	
		HR (95%CI)	*p* value		HR (95%CI)	*p* value
Gender (female/ male)	0.152			0.286		
Age (≤ 60/ > 60 years)	0.770			0.910		
Blood Loss (≤ 400 ml / > 400 ml)	**0.009**		0.499	**0.003**		0.316
Cirrhosis (yes/no)	0.060			**0.037**	1.703 (1.166–2.489)	**0.006**
Tumor number (single/multiple)	**0.006**	1.467 (1.027–2.097)	**0.035**	**0.002**	1.541 (1.092–2.176)	**0.014**
Tumor size (≤ 5 cm/ > 5 cm)	**0.022**	1.537 (1.023–2.310)	**0.039**	**0.004**	1.504 (1.009–2.242)	**0.045**
Differentiation (W + M/P)	** < 0.001**	2.025 (1.416–2.894)	** < 0.001**	**0.020**		0.127
Tumor stage (I + II/III + IV)	**0.011**		0.791	**0.026**		0.398
Resection Margin (R0/ R1)	**0.001**		0.520	**0.002**		0.632
TNM (I + II/III + IV + V)	** < 0.001**	2.211 (1.553–3.148)	** < 0.001**	** < 0.001**	2.013 (1.424–2.845)	** < 0.001**
CEA (≤ 5ug/l/ > 5 ug/l)	**0.009**		0.159	**0.001**	1.539 (1.102–2.148)	**0.011**
CA199 (≤ 35U/ml/ > 35)	0.263			0.399		
PT (≤ 13 s/ > 13 s)	0.261			0.656		
PLT (≤ 300 × 10^9/L/ > 300 × 10^9/L)	0.996			0.745		
Neutrophil (≤ 6.4 × 10^9/L/ > 6.4 × 10^9/L)	0.332			0.089		
Lymphocyte (≤ 3.3 × 10^9/L/ > 3.3 × 10^9/L)	0.352			0.299		
TBIL (≤ 22umol/L/ > 22 umol/L)	0.433			0.435		
DBIL (≤ 7umol/L/ > 7 umol/L)	0.605			0.596		
ALB (≤ 35 g/L/ > 35 g/L)	0.385			0.246		
ALT (≤ 40U/L/ > 40 U/L)	0.763			0.659		
AST (≤ 37U/L/ > 37U/L)	0.730			0.453		
HBsAg (yes/no)	0.112			**0.014**	1.543 (1.107–2.151)	**0.011**

Bold marks indicated that the results were statistically significant (*P* < 0.05)

*ICC* intrahepatic cholangiocarcinoma, *HBsAg* hepatitis B surface antigen, *W + M* well + moderately differentiated, *P* poorly differentiated, *TNM* tumor node metastasis, *CEA* carcinoembryonic antigen, *CA19-9* carbohydrate antigen 19-9, *NA* not applicable, *NS* not significant

### HBsAg expression was positive in HBV-positive ICC tissues

**Fig. 4:**
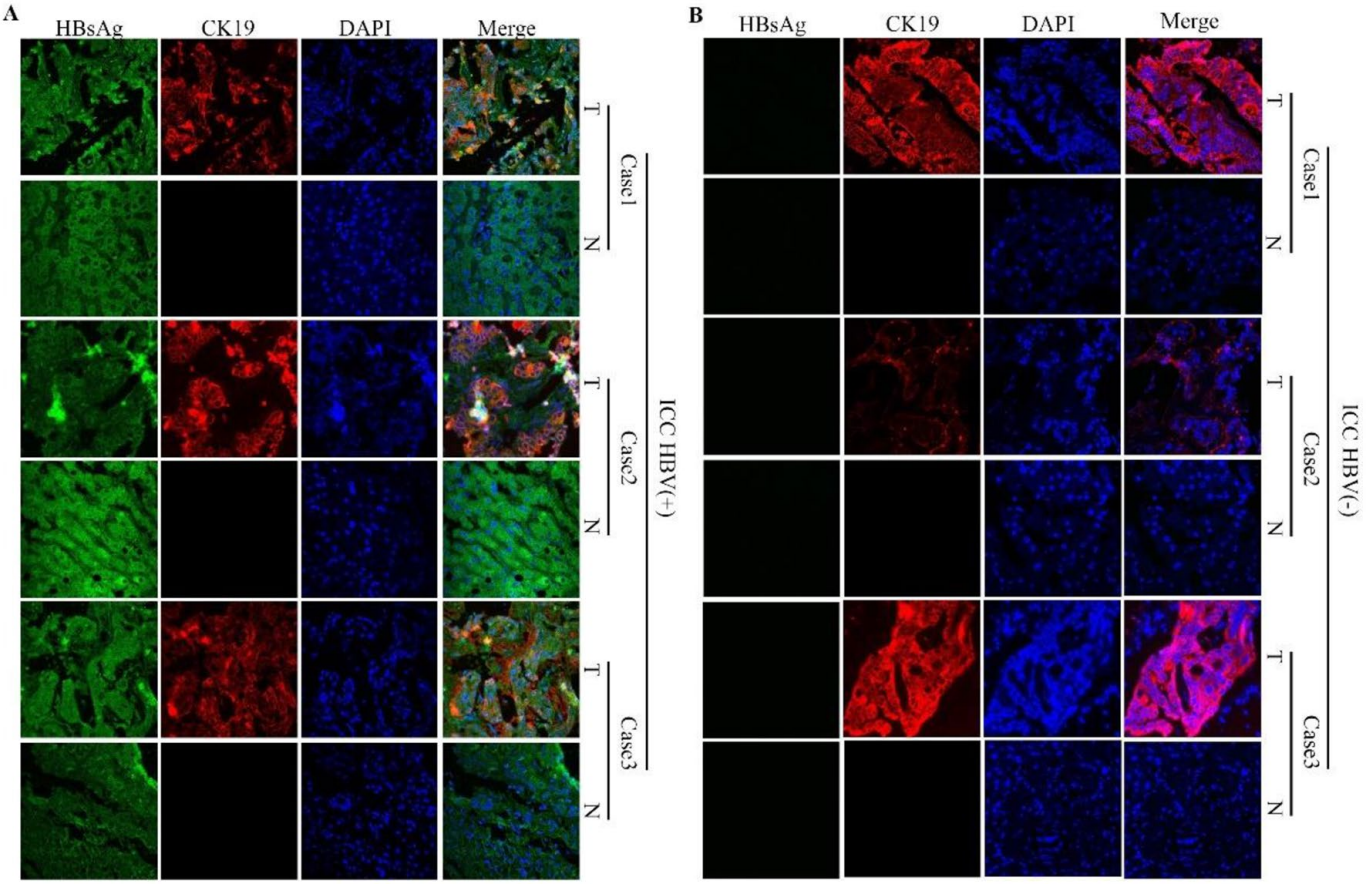
**a** IF assay results for paraffin sections of fresh ICC specimens and corresponding paracancer tissue specimens from 3 HBV-positive patients; **b** IF assay results of paraffin sections of fresh ICC specimens and corresponding paracancer tissue specimens from 3 HBV-negative

Fresh ICC specimens and corresponding paracancer tissue specimens were collected from 3 HBV-positive patients and 3 HBV-negative patients. These specimens were made into paraffin sections and subjected to IF staining. The results of IF staining are shown in Fig. 4, supplementary Fig. 1, supplementary Fig. 2 and supplementary Fig. 3. HBsAg was expressed in HBV-positive ICC tissues (100%, 3/3), but HBsAg was not expressed in HBV-negative ICC tissues (0%, 0/3). The results of IF staining also showed that CK19 and CK7 were expressed only in ICC tumor tissues (100%, 3/3), but not in adjacent nontumor tissues (0%, 0/3). As a negative control, results also showed that ALB (Supplementary Fig. 2) and Hep-Par1 (Supplementary Fig. 3) were only expressed in adjacent non-tumor tissue (100%, 3/3) and not in ICC tumor tissue (0%, 0/3).

In addition,we collected 3 HBV (+) ICC tissues and 3 HBV (-) ICC tissues respectively, constructed paraffin sections, and performed HE staining. The staining results are shown in supplementary Fig. 6. We found that the tumor cell growth of HBV (−) ICC was vascular-shaped and grew along the bile duct. The distribution of HBV (+) ICC tumor cells was relatively scattered and didn’t show vascular shape. The difference in HE staining results between HBV (+)ICC and HBV (−)ICC suggested that their origins might be different.

**Fig. 5:**
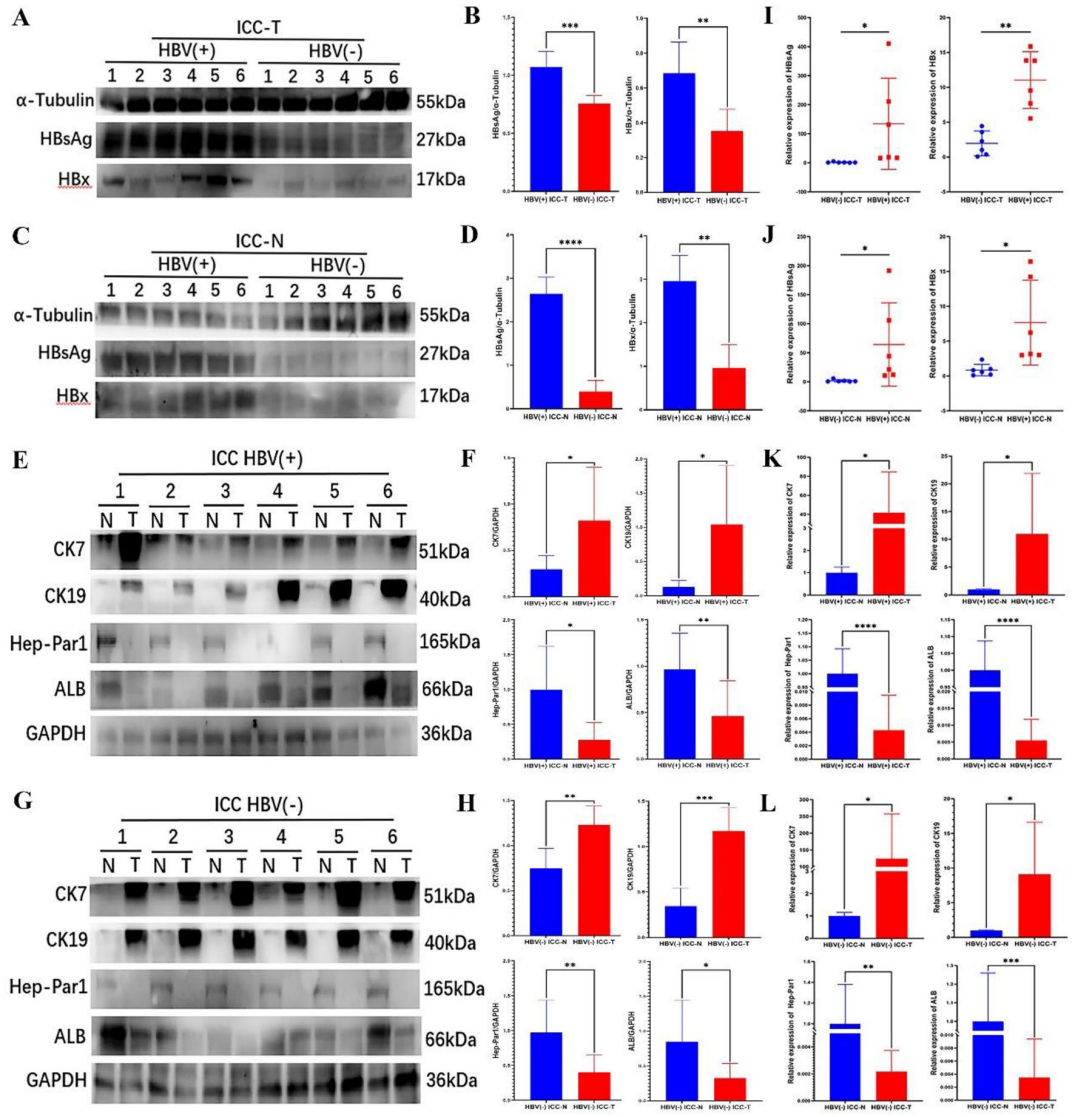
The expression of HBV-related genes (HBsAg and HBx), bile duct cell related genes (CK19 and CK7) and hepatocyte related genes (Hep-Par1and ALB) and corresponding proteins of these genes were verified

We collected tumor tissues (ICC-T) and corresponding paracarcinoma tissues (ICC-N) from 6 pairs of HBV (+) ICC patients and 6 pairs of HBV (−) ICC patients, respectively, extracted RNA and protein from the tissues, and then conducted RT-PCR and WB experiments. The expression of HBV-related genes (HBsAg and HBx), bile duct cell-related genes (CK19 and CK7) and hepatocyte-related genes (Hep-Par1 and ALB) and corresponding proteins of these genes were verified. The experimental results are shown in the Fig. 5. It was shown that the expression of HBsAg and HBx (Fig. 5I) and corresponding protein (Fig. 5A) in HBV (+) ICC-T tissue was significantly higher than that in HBV (−)ICC-T tissue, and the same conclusion was found in ICC-N tissue (Fig. 5C, J). Meanwhile, the experimental results also showed that the expressions of biliary duct cell related genes (CK19 and CK7) (Fig. 5K) and corresponding proteins (Fig. 5E) in HBV (+) ICC-T tissue were significantly higher than those in HBV (+)ICC-N tissue. The expressions of hepatocyte related genes (Hep-Par1and ALB) (Fig. 5K) and corresponding proteins (Fig. 5E) were significantly lower than those of HBV (+)ICC-N tissue. Similar conclusions were found in HBV (−) ICC tumor tissues and corresponding paracancer tissues (Fig. 5G, L). The WB results were quantized using imageJ, and the results were shown as Fig. 5B, D, F, H respectively.

### In vitro organoid culture experiments showed that HBsAg staining was found only in HBsAg-positive ICC organoids, but not in negative

**Fig. 6:**
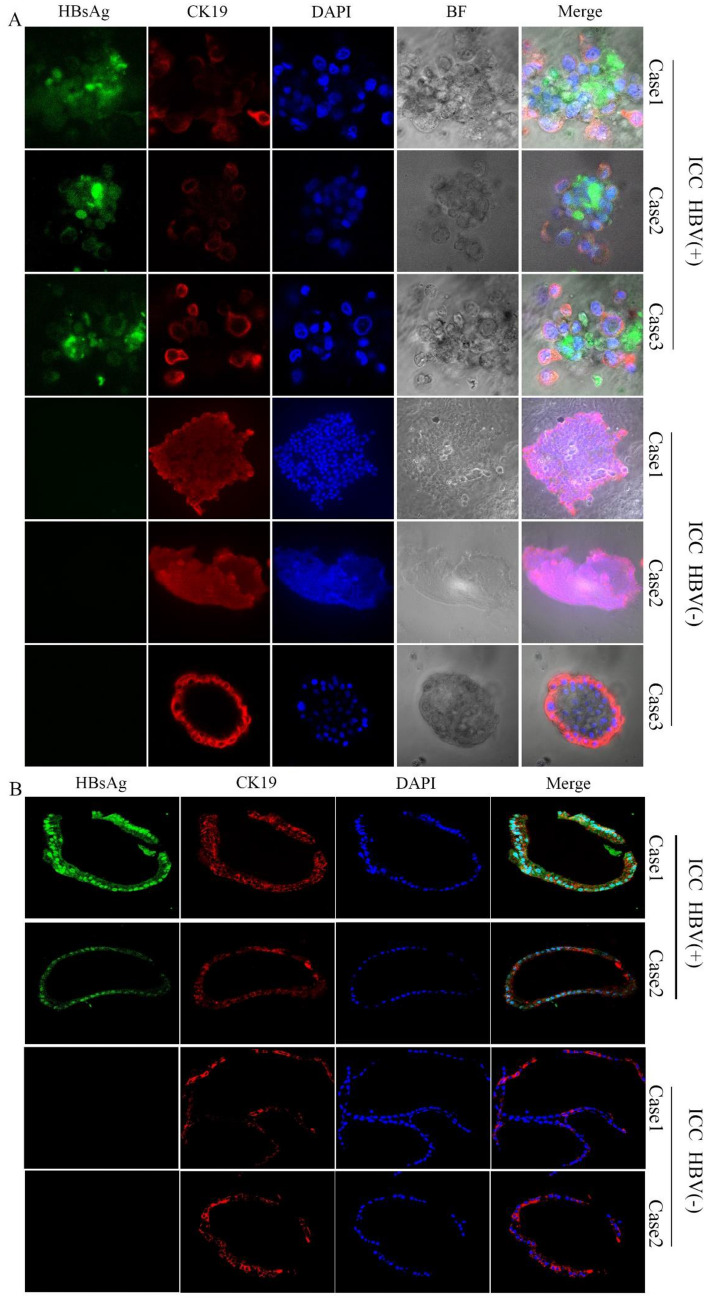
**a** the results of IF assay of 3 HBV ( +) organoids and 3 HBV (−) organoids. **b** Results of IF assay on paraffin sections of 2 HBV ( +) and 2 HBV (−) organoids

IF assays were performed on ICC tissue derived organoids. The detection indexes included HBsAg, CK19, CK7,DAPI and bright field (BF). Finally, several indicators were merged to obtain the merge field. Organoids from 5 pair of HBV ( +) and HBV (−) ICC patients were selected for IF experiment (Fig. 6). Among them, 3 pairs were directly stained with IF on organoids culture plates (Fig. 6). As for the other 2 pairs of organoids, paraffin sections of organoids were first prepared and then stained with IF (Fig. 6B). In addition, we also conducted IF experiments on 3 HBV (+) and 3 HBV (−) organoids paraffin sections,the staining indexes included HBsAg, CK7 and DAPI (Supplementary Fig. 5). The results showed that HBsAg staining was found only in HBsAg-positive ICC organoids (100%, 8/8), but not in negative ICC organoids (0%, 0/8).

### HBV-DNA could be detected in the medium of HBV-positive organoids

**Fig. 7:**
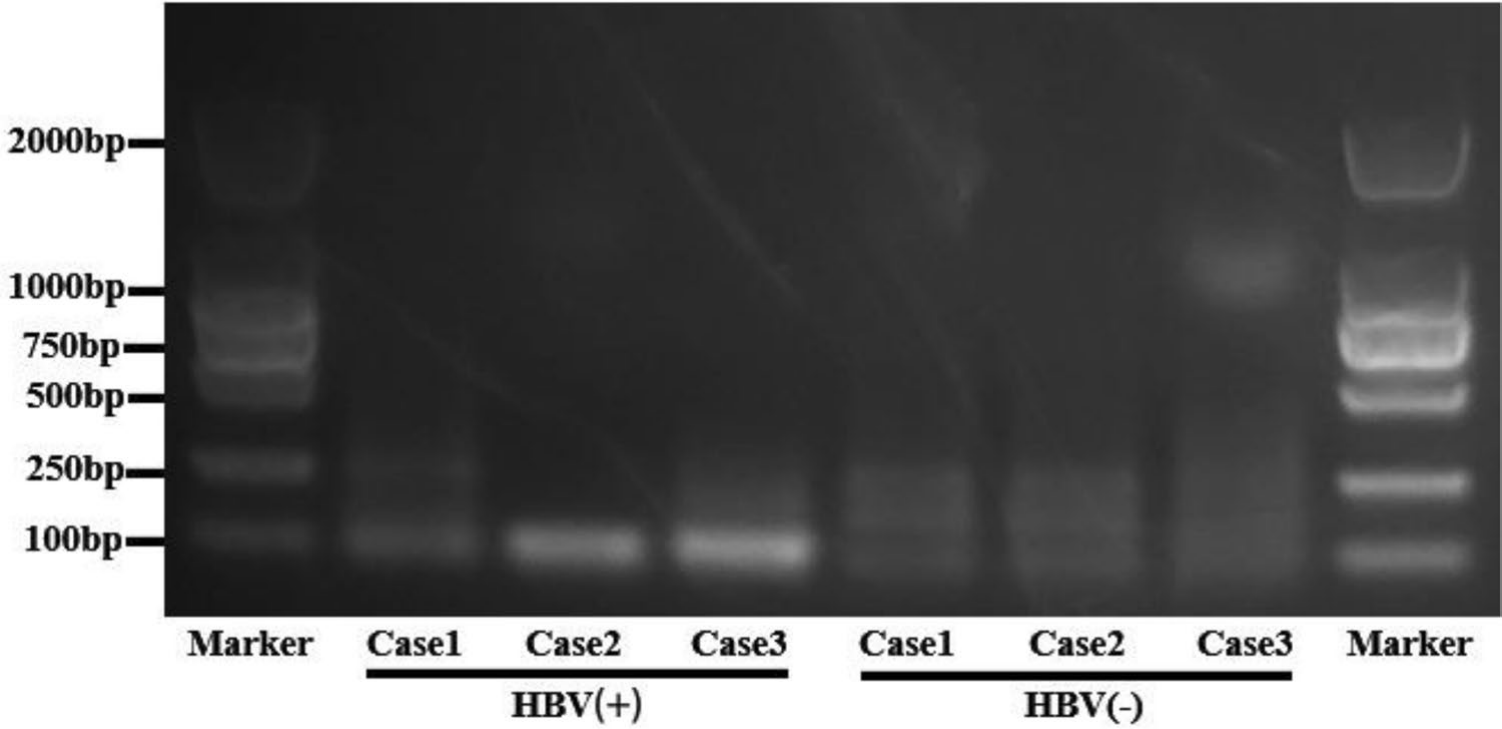
The result of PCR electrophoresis shown that HBV-DNA was present in the supernatant of 3 HBV ( +) ICC derived organoids, while no HBV-DNA was present in the supernatant of 3 HBV (−) ICC derived organoids

HBV-DNA could be detected in the culture medium of HBV ( +) ICC derived organoids in all three cases, which were 1.528 × 10^2 IU/mL, 2.515 × 102 IU/mL, 1.450 × 103 IU/mL, respectively. In contrast, HBV-DNA was not detected in the culture medium of HBV (−) ICC-derived organoids in the three cases, as shown in Table 3. The result of PCR electrophoresis is shown in Fig. 7. HBV-DNA was present in the supernatant of 3 HBV (+) ICC derived organoids, while no HBV-DNA was present in the supernatant of 3 HBV (−) ICC derived organoids.

**Table 3:** Results of HBV-DNA titer measurement in culture medium of ICC derived organoids from three HBV (+) cases and three HBV (−) cases

Variables	HBV ( +)			HBV (−)		
	Case1	Case2	Case3	Case1	Case2	Case3
Culture medium concentration (IU/mL)	1.528 × 10^2	1.450 × 10^3	2.515 × 10^2	0	0	0
Serological concentration (IU/mL)	< 100	< 100	< 100	< 100	< 100	< 100
HBsAg concentration (IU/mL)	92.78	3576.96	1192.32	0	0	0
HBsAg ( ±)	** + **	** + **	** + **	**−**	**−**	**−**
HBsAb ( ±)	**−**	**−**	**−**	**−**	** + **	** + **
HBeAg ( ±)	**−**	**−**	**−**	**−**	**−**	**−**
HBeAb ( ±)	** + **	** + **	** + **	**−**	**−**	**−**
HBcAb ( ±)	** + **	** + **	** + **	** + **	** + **	** + **

Bold positive and negative markers are used to better highlight the results

### There was no expression of HBV in the bile duct cells in the portal area

**Fig. 8:**
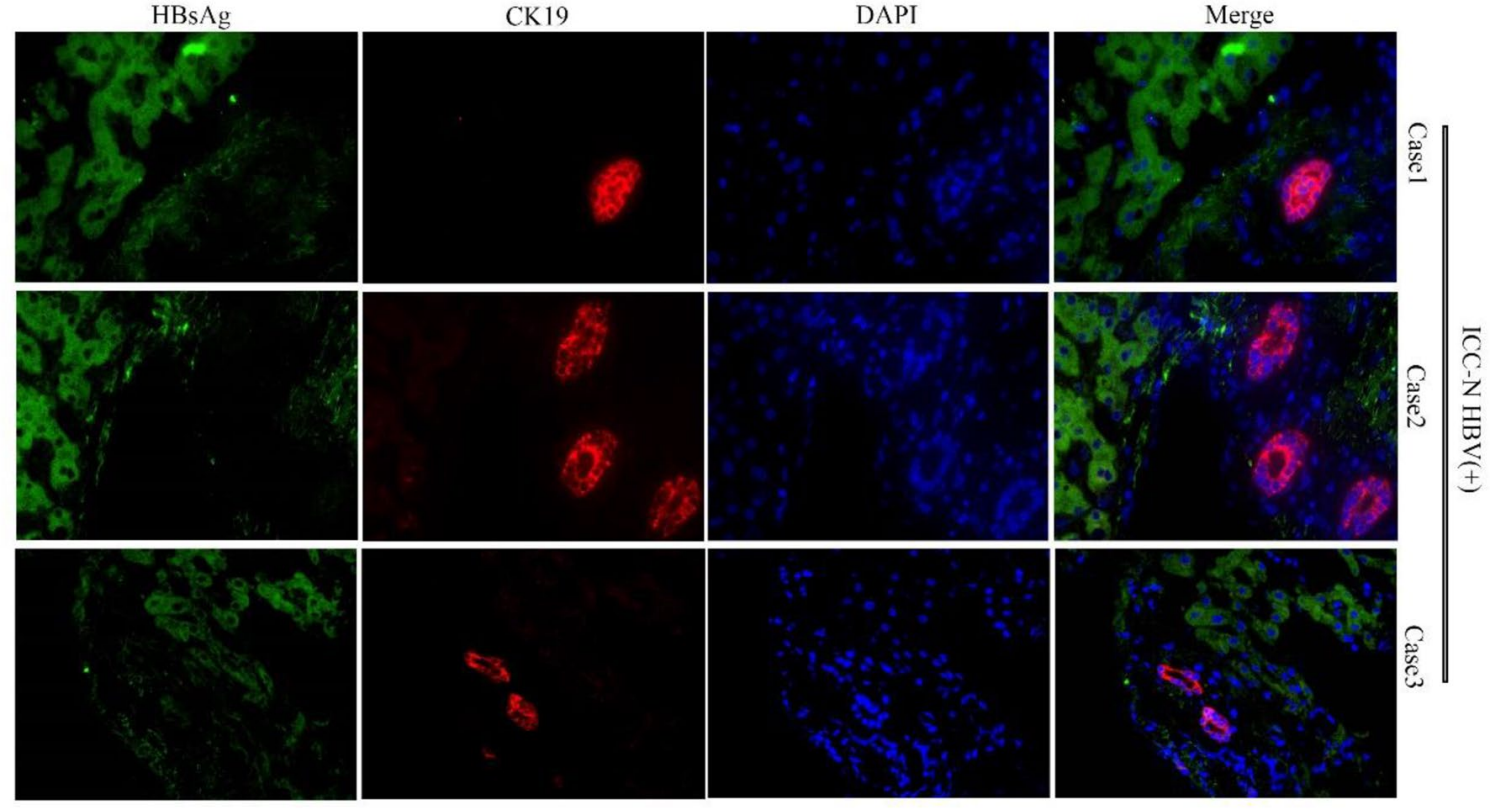
CK19 was expressed in the bile duct cells in the portal area. HBsAg was expressed in hepatocytes, but not in the bile duct cells

**Fig. 9:**
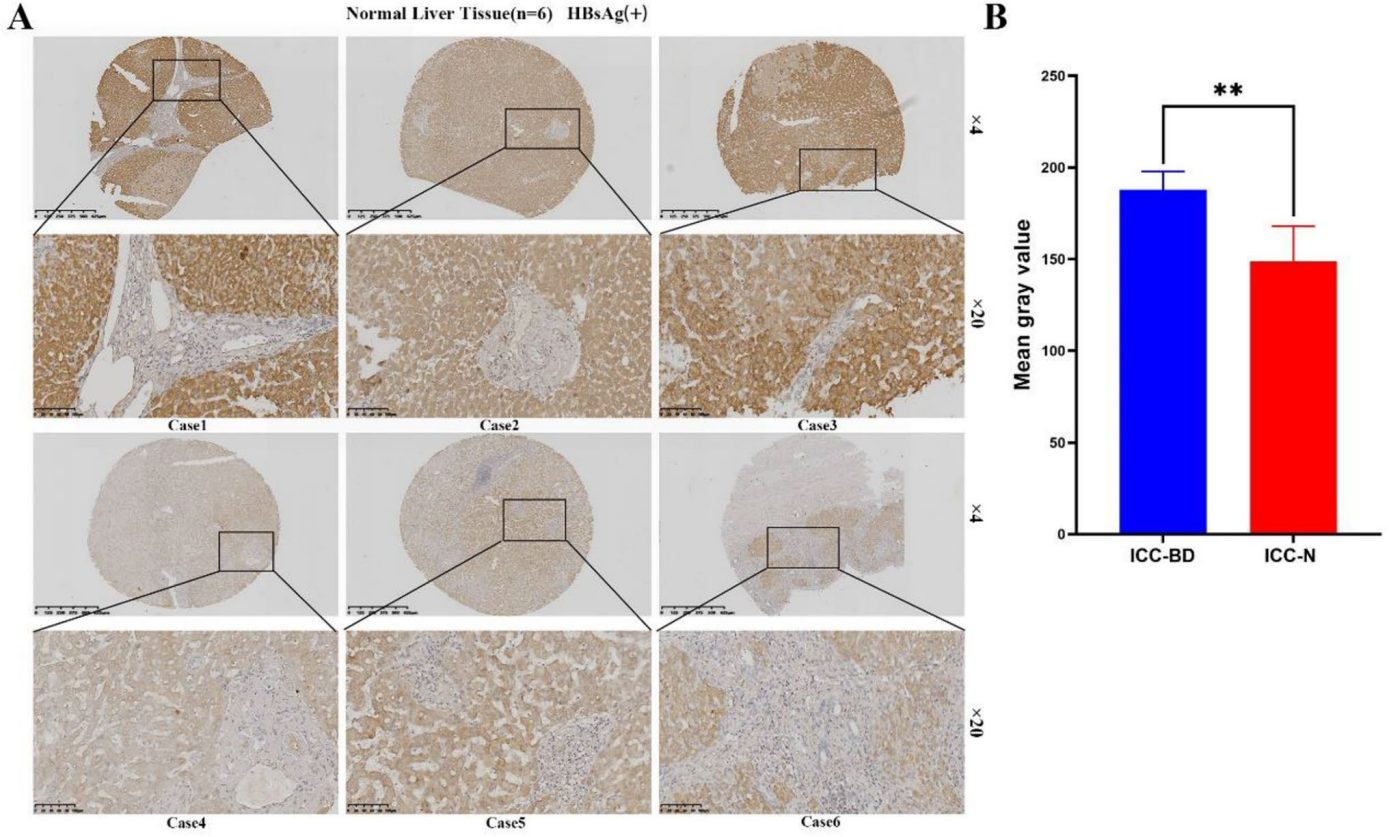
**a** The results of IHC experiments on tissue array found that HBs antigen is only expressed in hepatocytes of normal tissues, but not in intrahepatic bile duct epithelial cells. IHC results of 6 HBV ( +) non-ICC patients were selected. **b** The IHC results were quantized by imageJ

**Fig. 10:**
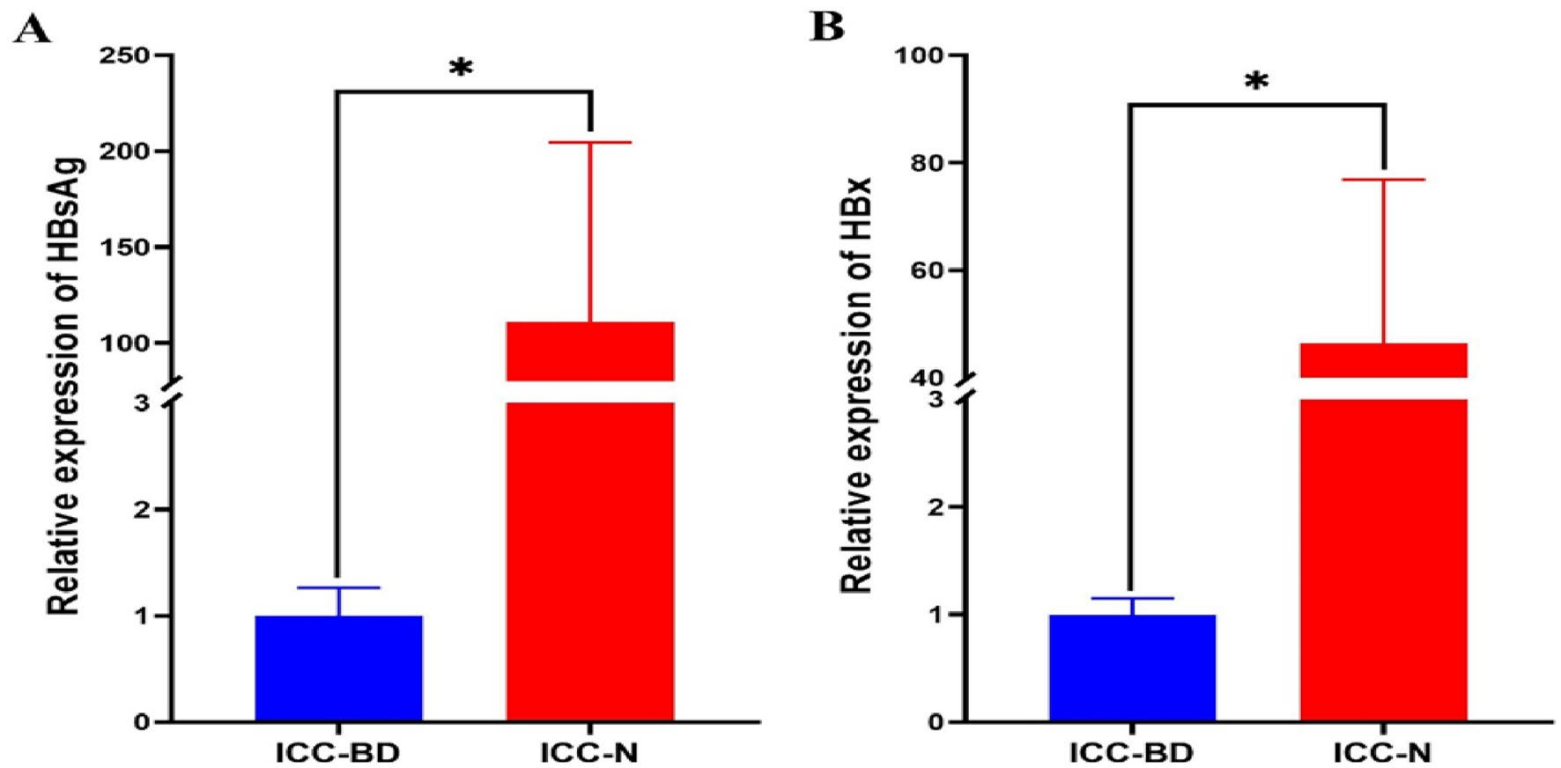
Quantitative PCR assay showed that the expression of HBs antigen and HBx in normal hepatocytes (ICC-N) were significantly higher than that in bile duct epithelial cells (ICC-BD)

IF staining of paraffin sections of adjacent nontumor tissues (HBV (+) ICC-N) was used to further determine whether bile duct epithelial cells in the portal vein area were infected with HBV. The procedure was the same as that of IF assay in paraffin sections of ICC tumor tissue (HBV (+) ICC-T). We confirmed the location of bile duct cells through positive expression of CK19 and CK7. We found HBsAg expression was negative in the bile duct cells in the portal area, indicating no HBV infection in this group of cells. (Fig. 8 and Supplementary Fig. 6). By conducting IHC experiments on tissue array, we found that HBs antigen was only expressed in hepatocytes in normal tissues, but not in intrahepatic bile duct epithelial cells. Figure 9 shows the IHC results of 6 HBV (+) non-ICC patients. In addition, we collected adjacent nontumor tissues of 6 patients with HBV (+) ICC, from which biliary duct tissue and normal liver tissue were isolated and RNA was extracted, respectively, for quantitative PCR assay. As shown in the Fig. 10, the expression of HBs antigen (Fig. 10A) and HBx (Fig. 10B) in normal hepatocytes (ICC-N) were significantly higher than that in bile duct epithelial cells (ICC-BD). Combined with the IF and IHC staining, it was confirmed that HBV does not infect normal bile duct epithelial cells.

### There was no difference in the expression of ICC associated conventional and tumor suppressor oncogenes in HBV (+) ICC and HBV (−) ICC

**Fig. 11:**
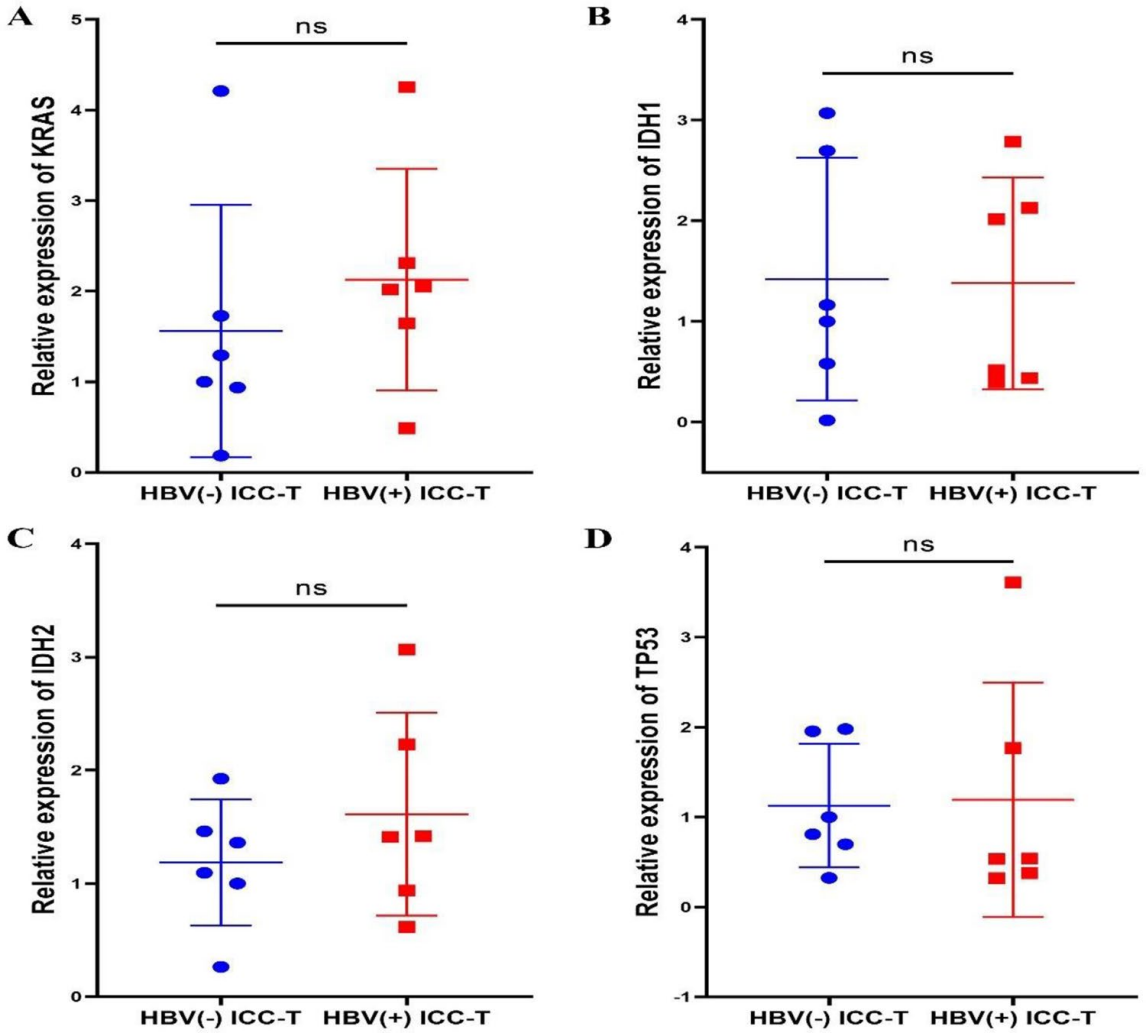
Quantitative PCR assay showed that there was no significant difference in the expression of oncogenes KRAS (**a**), IDH1 (**b**) and IDH2 (**c**), and tumor suppressor gene TP53 (**d**) between HBV ( +) ICC and HBV (−) ICC

We collected tumor tissues from 6 patients with HBV (+) ICC and 6 patients with HBV (-) ICC. RNA was extracted and then reverse-transcribed to form cDNA. Then qPCR experiment was conducted, and the detection indicators included oncogenes KRAS, IDH1 and IDH2, and tumor suppressor gene TP53. As shown in Fig. 11, there was no significant difference in the expression of oncogenes KRAS (Fig. 11A), IDH1 (Fig. 11B) and IDH2 (Fig. 11C), and tumor suppressor gene TP53 (Fig. 11D) between HBV ( +) ICC and HBV (−) ICC.

## Discussion

In this study, we found that HBV infection is present in as high as 40.66% ICC patients. Then, through IHC and IF staining on tissue microarray and organoid tissues, we found that HBsAg was positive only on ICC tissues (and organoids) from HBV-infected ICC patients. Likewise, we found the expression of HBV-DNA in supernatants only from organoids derived from HBV-infected ICC patients. There are also some evidences that mature hepatocytes retain phenotypic plasticity and can differentiate into bile duct cells in vitro and in vivo [30–33]. These results give us a strong proof that HBV lead to the transdifferentiation of hepatocytes into ICC cells. There is a causal relationship between HBV infection and ICC formation.

HBV infection is one of the main causes of ICC [34]. One study showed that preoperative antiviral therapy effectively reduced the risk of postoperative viral reactivation in HBV-positive ICC patients, thus prolonging the long-term survival of ICC patients [35]. Postoperative antiviral therapy can also achieve the same goal. In contrast, other researchers suggested that HBV had no effect on ICC survival [36]. Furthermore, some researchers have found that HBV infection or preoperative HBV vaccination are favourable factors for survival, which can improve the survival rate of patients with ICC surgery [37, 38]. Our study showed that the DFS rate of ICC patients in HBsAg (+) group was significantly lower than that of ICC patients in HBsAg (−) group (*p* = 0.0137), and HBsAg expression is an independent predictor of poor DFS in ICC patients (*p* = 0.011). The OS rate of ICC patients in HBsAg (+) group was lower than that in HBsAg (−) group, but the difference was not statistically significant (*p* = 0.1121). Although the difference in OS between the two groups was not statistically significant, it would be more pronounced with the increase in the number of cases. These results might provide evidence supporting the use of postoperative anti-HBV therapy to prevent ICC tumor recurrence.

The relationship between HBV infection and ICC survival is complex and requires further investigation [38]. First of all, the pathogenesis of HBV infection-related ICC may be similar to that of HCC [39]. As one of the most common risk factors of ICC, HBV can integrate HBV genome fragments into the genome of target cells and lead to cell transformation. On the other hand, it can select hepatocytes or bile duct cells with malignant tendencies, thus leading to the development of tumors [40]. The similar process of infection and carcinogenis makes the clinical characteristics of HBV (+) ICC similar to that of HBV (+) HCC. Similarly, HBV (+) ICC patients have a worse prognosis, which is consistent with the conclusion of this study. Contrary to this, some studies have shown that HBV (+) ICC patients have a better prognosis. They surmised that HBV infection can activate innate and acquired immune responses in patients with a history of HBV infection, thus enhancing the anti-tumor activity of ICC patients and benefiting the survival of ICC patients. [41, 42].

Previous studies have shown that tumor number [43], tumor size [44], TNM stage [45], cirrhosis [46], CEA [47] and tumor differentiation [48] were closely related to the survival rate of ICC patients and can predict the survival of ICC patients. This study also showed that tumor number (*p* = 0.014), tumor size (*p* = 0.045), TNM stage (*p* < 0.001), cirrhosis (*p* = 0.006) and CEA (*p* = 0.011) were independent predictors of DFS in ICC patients. In addition, tumor number (*p* = 0.035), tumor size (*p* = 0.039), TNM stage (*p* < 0.001) and tumor differentiation (*p* < 0.001) were independent predictors of OS in ICC patients. These indicators of clinical significance can be further improved by increasing the number of cases in the future, and then a prediction model can be constructed to predict the prognosis of ICC patients more precisely.

Previous study showed that preoperative elevated serum neutrophil–lymphocyte ratio (NLR) was an independent risk factor for OS and tumor recurrence in HBV (+) ICC patients [49]. In addition, a number of studies have also shown that increased NLR predicts early tumor recurrence, high recurrence rate and shorter survival in ICC patients [50, 51]. In this study, we found that compared with HBsAg (-) patients, HBsAg (+) patients had higher neutrophil cell count and higher corresponding NLR (*p* = 0.005), which was consistent with the conclusion of the above study.

Hepatitis B virus, as a hepatophilic virus, generally attacks only liver cells [15, 16, 52], so HBsAg is usually expressed only in hepatocytes. By IF staining of fresh ICC tissues and corresponding paracancer tissues, we found HBsAg expression in HBV (+) ICC tissues, and distinguished ICC tissues from corresponding paracarcinoma tissues by CK19 and CK7 expression. In addition, to exclude the possibility that the HBsAg expression in HBV (+) ICC tissues was due to the mixture of normal hepatocytes, we prepared organoids from ICC tissues and proved that HBsAg was positive only in organoids from HBV (+) ICC tissues. HBsAg acts as a "tracer protein", strongly supporting that ICC may be derived from hepatocytes. Combined with the difference in HE staining results between HBV (+) ICC tissue and HBV (-) ICC tissue, this evidence indicates that HBV-associated ICC may originate from hepatocytes. The expression of oncogenes KRAS, IDH1, IDH2 and tumor suppressor gene TP53 showed no difference in HBV (+) ICC and HBV (-) ICC tumor tissues, indicating that there was no significant differences in gene mutations between the two ICC with different origins.

HBsAg staining is usually negative in HBV (+) HCC tissues [53]. The IHC experiment of tumor tissues of patients with HBV (+) HCC showed that the positive rate of HBsAg in normal liver tissues around HCC was significantly higher than that in tumor tissues. HBsAg in normal liver tissue is usually strongly expressed in the cytoplasm of liver cells. However, in tumor tissues, HBsAg is usually weakly positive on the cell membrane of tumor cells [53]. Our study showed that HBsAg expression in HBV (+) ICC tumor cells and normal liver cells were all located in the cytoplasm. Therefore, the expression of HBsAg in HCC and ICC tumor cells is different.

A previous study demonstrated that HBV integration is a common event in HBV-related ICC by detecting ICC tissues and corresponding paracancer tissues through high-throughput capture sequencing method [54]. There are two possible explanations for this integration: one is that HBV is directly integrated into ICC tumor cells, the other is that HBV is first integrated into hepatocytes and then further induced malignant transformation of hepatocytes into ICC. As there is no mature animal model for relevant experiments at present, there is no way to conduct lineage tracing experiment to confirm that ICC was transformed from hepatocytes, which is also the shortcoming of our experiment. However, the fact that we were able to detect HBV-DNA expression in the supernatant of organoid cultures suggests that this integration is functional. Since HBV is hepadnavirus and only infect hepatocytes, we may spectulate that the second explanation is more reasonable.

Previous studies have shown that HBV may infect bile duct cells [55, 56]. By IF staining of paracancer tissue, we observed that there was no HBsAg expression in bile duct cells in the portal area of paracancer tissue in 6 ICC patients (Fig. 8 and Supplementary Fig. 6)**,** thus the possibility of HBV infection of bile duct cells could be ruled out. It has been suggested that a small number of interstitial cells, especially those in the portal area, can also be infected by HBV [55, 57]. To eliminate the interference of interstitial cells, we cultured organoids from ICC tissues and corresponding paratumor tissues, respectively, and then carried out IF staining on the organoids and detected the HBV-DNA level in the organoid culture medium. The same conclusion was reached by IF staining of organoids. Only the organoids derived from HBV (+) ICC tissue showed HBsAg positive staining, while the organoids derived from HBV (−) ICC tissue showed HBsAg negative staining.

In line with this phenomenon, we detected high levels of HBV-DNA in the culture medium of HBV (+) organoids, but not in the culture medium of HBV (−) organoids, which proved that HBV (+) ICC tissue-derived organoids had continuous replication and expansion of HBV virus. HBV can survive in ICC tumor cells derived from hepatocytes.

## Conclusion

In conclusion, we found that HBV-related ICC patients had shorter DFS than ICC patients with no HBV infection. HBV-related ICC might be derived from hepatocytes.

### Supplementary Information

Below is the link to the electronic supplementary material.

Supplementary file1 (DOCX 2488 KB)

## Data Availability

The data used or analyzed during this study are included in this article and available from the corresponding author upon reasonable request.
