# Supplementary material for: Hepatitis B virus-related intrahepatic cholangiocarcinoma originates from hepatocytes
Source: Hepatol Int. 2023 Jun 27;17(5):1300–17. doi: 10.1007/s12072-023-10556-3 (PMC10522522; doi:10.1007/s12072-023-10556-3)
Supplement: Supplementary file 1 — Supplementary file1 (DOCX 2488 KB) [file 12072_2023_10556_MOESM1_ESM.docx]

| **Supplementary results:** | |
| --- | --- |
| **Names** | **Sequences** |
| HBsAg- Forward Primer | 5`-TTGGTGTCTTTCGGAGTGTGGATTC-3` |
| HBsAg- Reverse Primer | 5`-GCCTCGTCGTCTAACAACAGTAGTC-3` |
| HBx- Forward Primer | 5`-AGCAATGTCAACGACCGACCTTG -3` |
| HBx- Reverse Primer | 5`-GACCAATTTATGCCTACAGCCTCCTAG-3` |
| CK19- Forward Primer | 5`-ACGGCGAGCTAGAGGTGAAGATC-3` |
| CK19- Reverse Primer | 5`-TGGTCGTGTAGTAGTGGCTGTAGTC-3` |
| CK7- Forward Primer | 5`-AGTATGAGGAGATGGCCAAATG-3` |
| CK7- Reverse Primer | 5`-CTGGTTCTTGATGTTGTCGATC-3` |
| Hep-Par- Forward Primer | 5`-CACAGAGGTCATCAAGGCAGAACAG -3` |
| Hep-Par- Reverse Primer | 5`-GCATAGGCGGAACGGATCATCAC -3` |
| ALB- Forward Primer | 5`-AAACATGTGTTGCTGATGAGTC-3` |
| ALB- Reverse Primer | 5`-TTCACCATAGGTTTCACGAAGA-3` |
| KRAS- Forward Primer | 5`-CATCTCCCAGGTTCAAGCGATTCTC-3` |
| KRAS- Reverse Primer | 5`-TGTGGTGACTGGCATCTGGTAGG-3` |
| IDH1- Forward Primer | 5`-CCAACTCTTCGCCAGCATATCATCC-3` |
| IDH1- Reverse Primer | 5`-TCTCTACCACAGAACCGCCACTG-3` |
| IDH2- Forward Primer | 5`-TGGAGAAGGTGTGCGTGGAGAC-3` |
| IDH2- Reverse Primer | 5`-GGTGTTCAGGAAGTGCTCGTTCAG-3` |
| TP53- Forward Primer | 5`-GGTTTCCGTCTGGGCTTCTTGC-3` |
| TP53- Reverse Primer | 5`-ATGTGCTGTGACTGCTTGTAGATGG-3` |
| GAPDH- Forward Primer | 5`-TGTGGGCATCAATGGATTTGG-3` |
| GAPDH- Reverse Primer | 5`-ACACCATGTATTCCGGGTCAAT-3` |

Supplementary Table 1: Primers used for RT-qPCR.


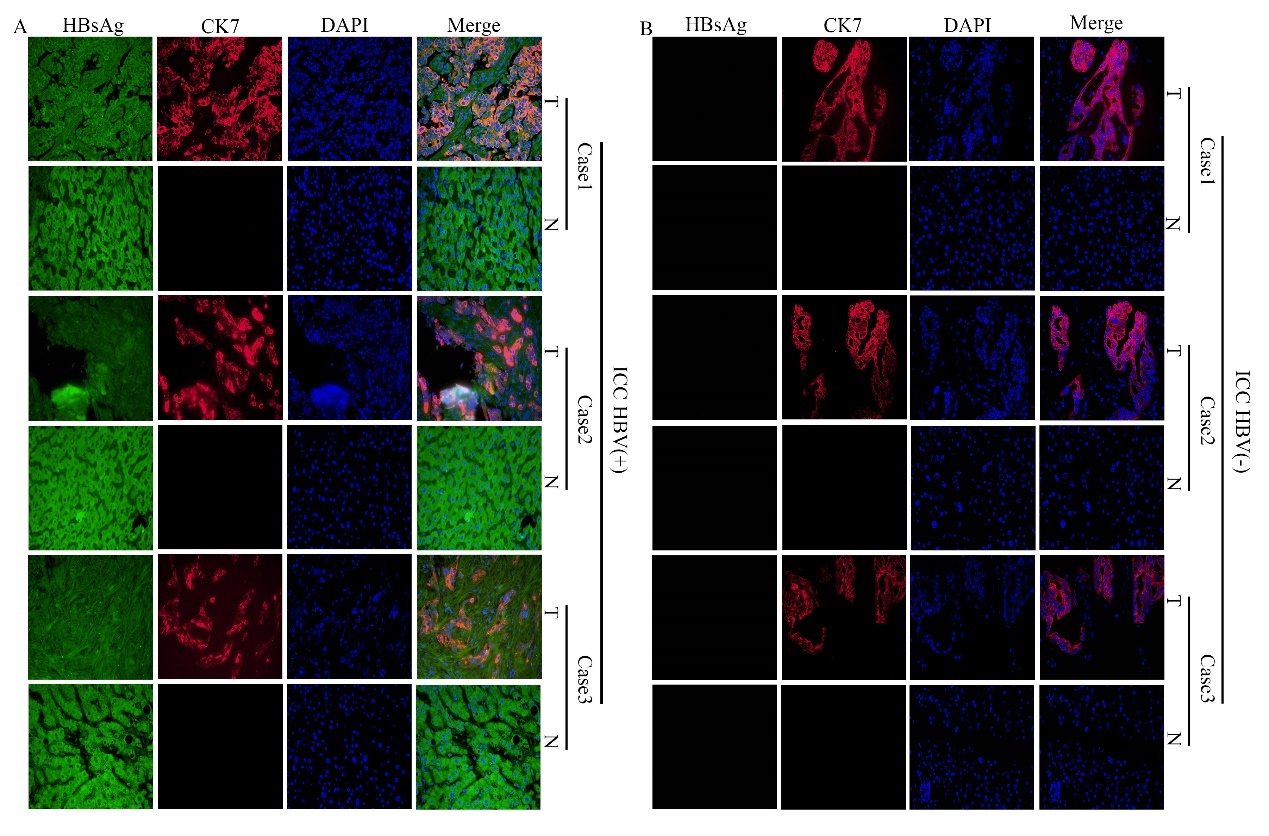


Supplementary Figure 1: IF assay results for paraffin sections of fresh ICC specimens and corresponding paracancer tissue specimens from 3 HBV-positive patients (A) and 3 HBV-negative patients (B). The staining indexes included HBsAg, CK7 and DAPI.


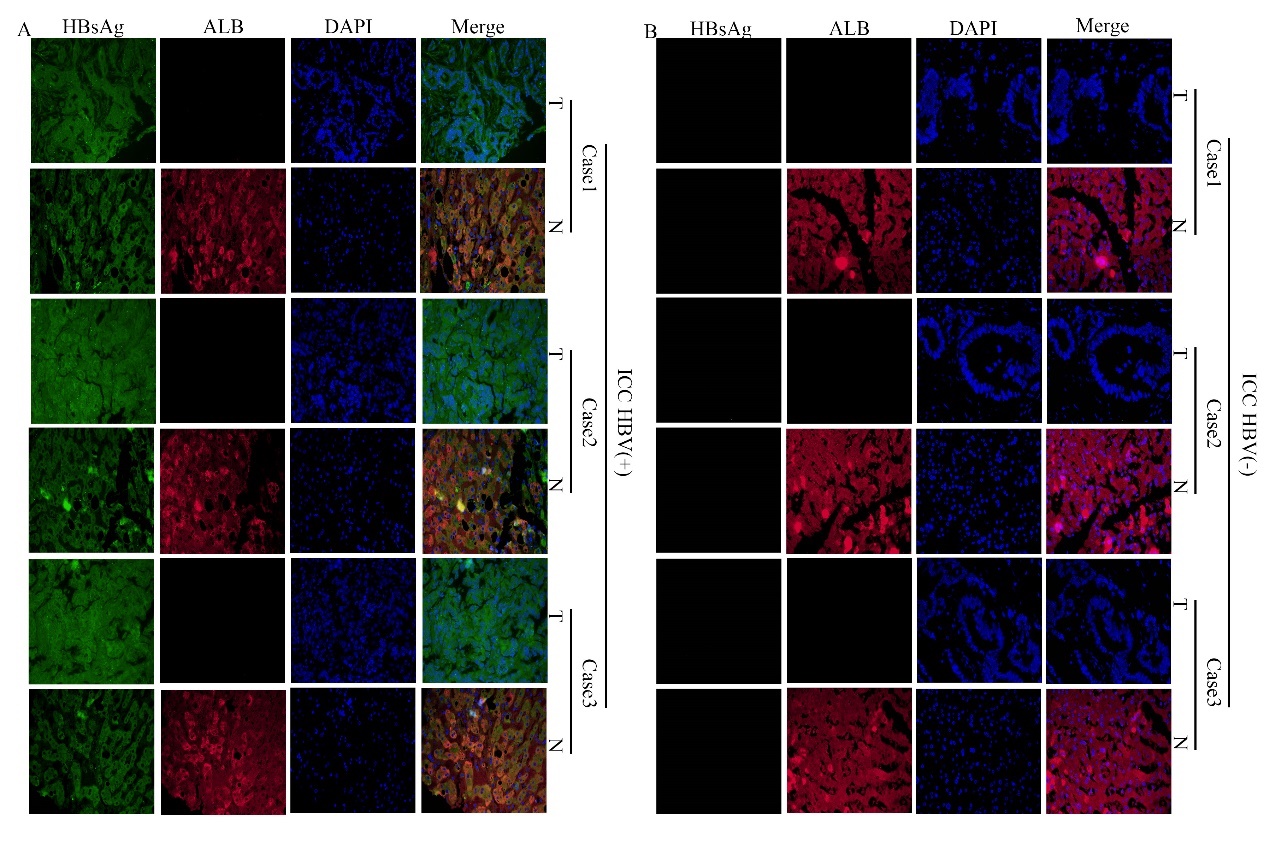


Supplementary figure 2: IF assay results for paraffin sections of fresh ICC specimens and corresponding paracancer tissue specimens from 3 HBV-positive patients (A) and 3 HBV-negative patients (B). The staining indexes included HBsAg, ALB and DAPI.

.


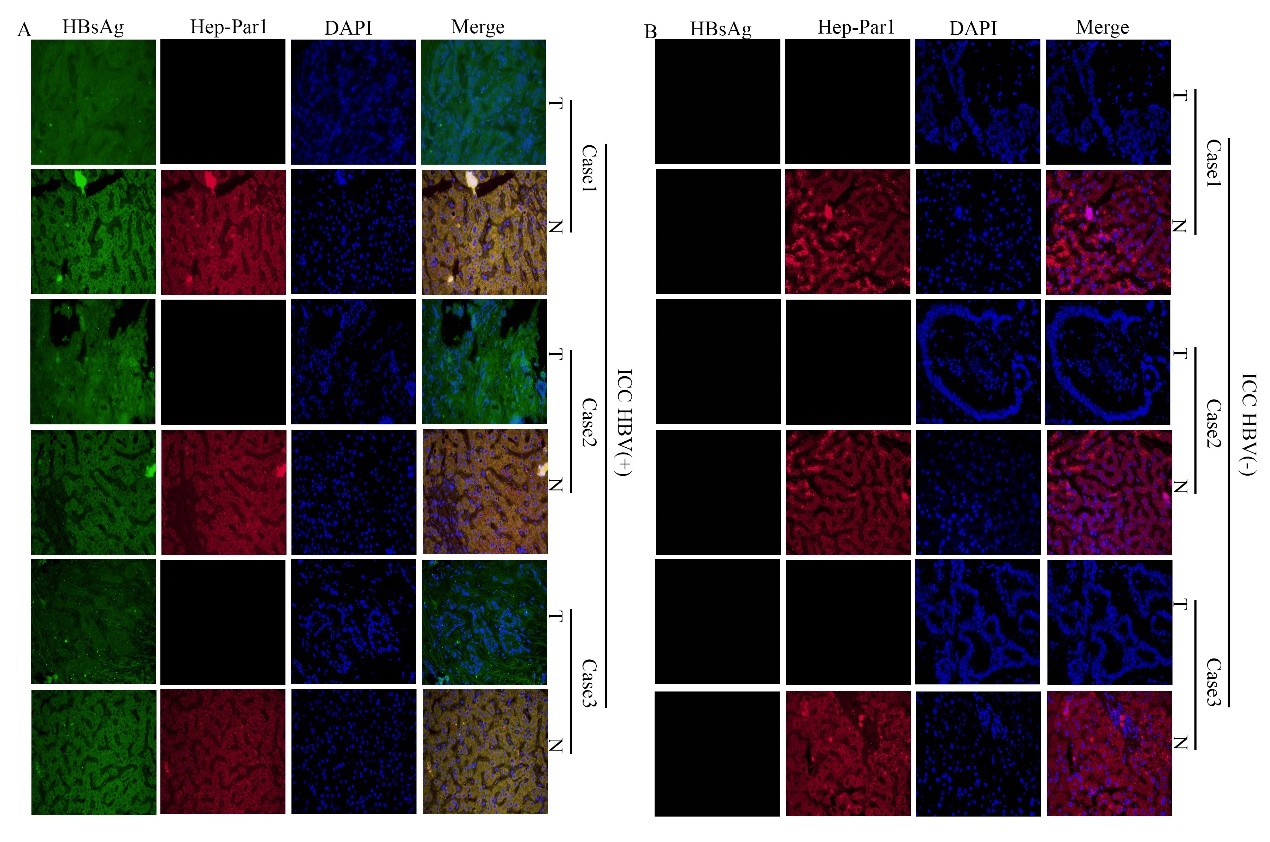


Supplementary figure 3:IF assay results for paraffin sections of fresh ICC specimens and corresponding paracancer tissue specimens from 3 HBV-positive patients (A) and 3 HBV-negative patients (B). The staining indexes included HBsAg, Hep-Par1 and DAPI.


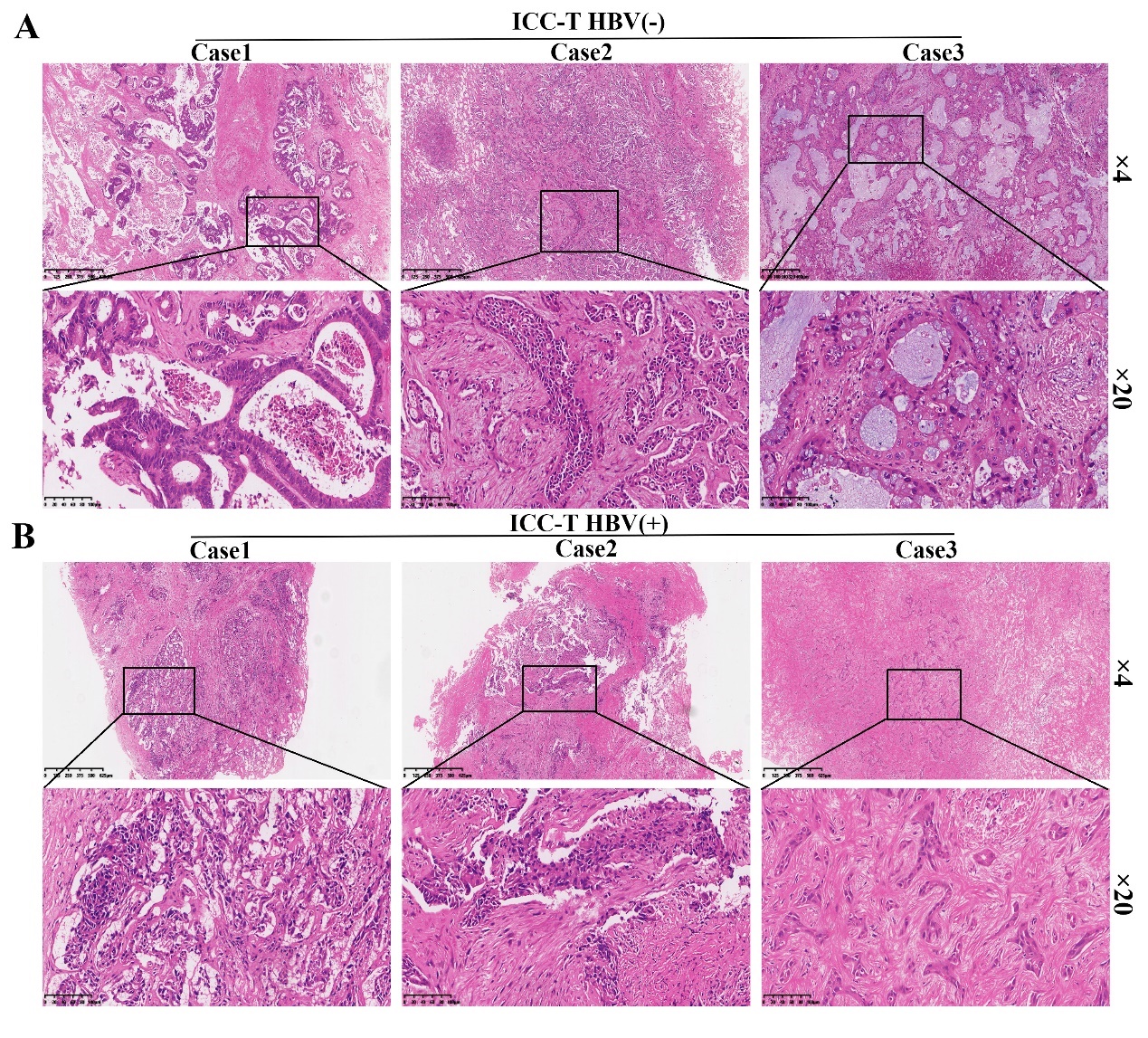


Supplementary figure 4: 3 HBV (+) ICC tissues and 3 HBV (-) ICC tissues were collected respectively, constructed paraffin sections, and performed HE staining.


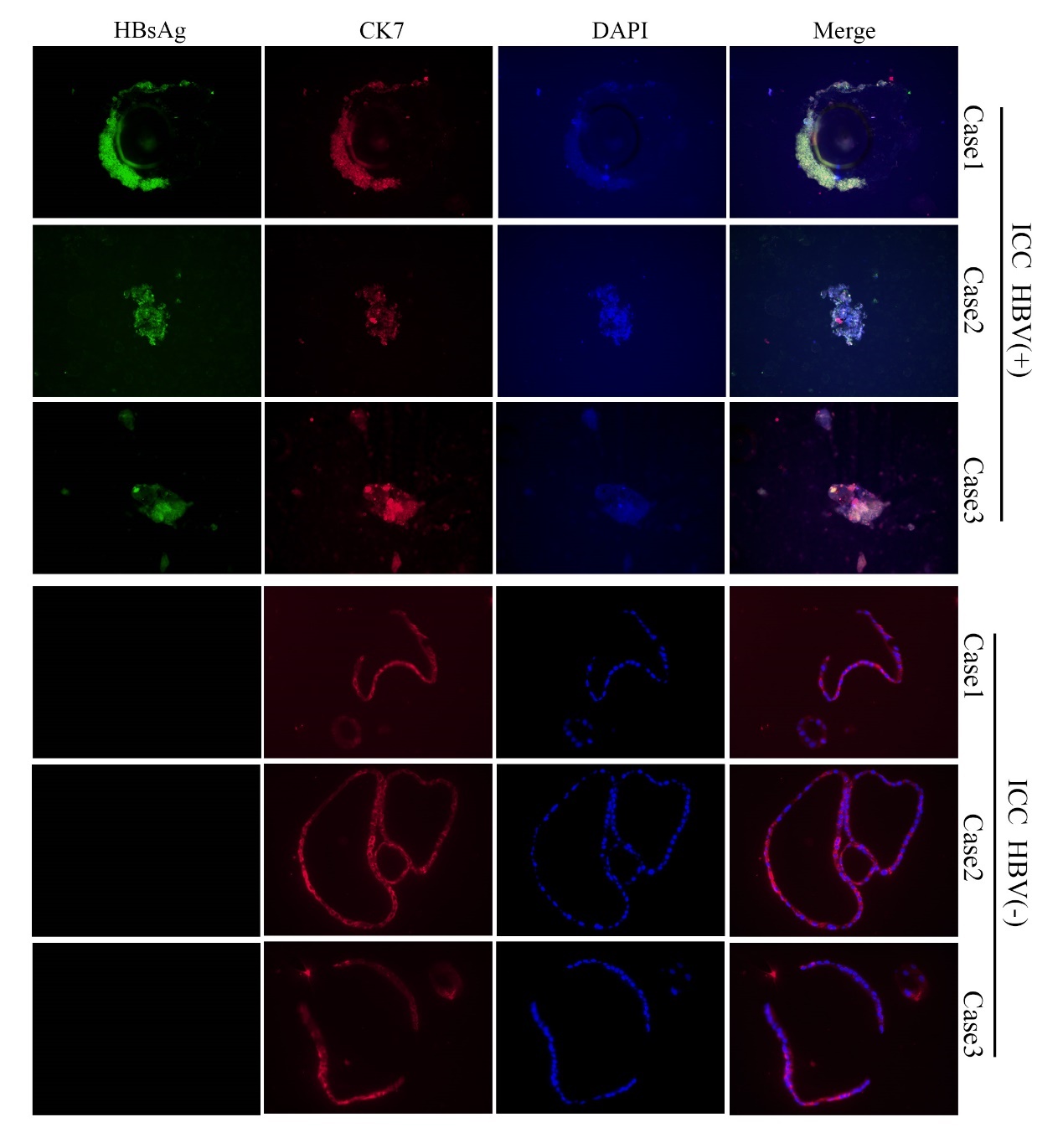


Supplementary figure 5: Results of IF assay on paraffin sections of 3 HBV(+) and 3 HBV(-) organoids. The staining indexes included HBsAg, CK7 and DAPI.


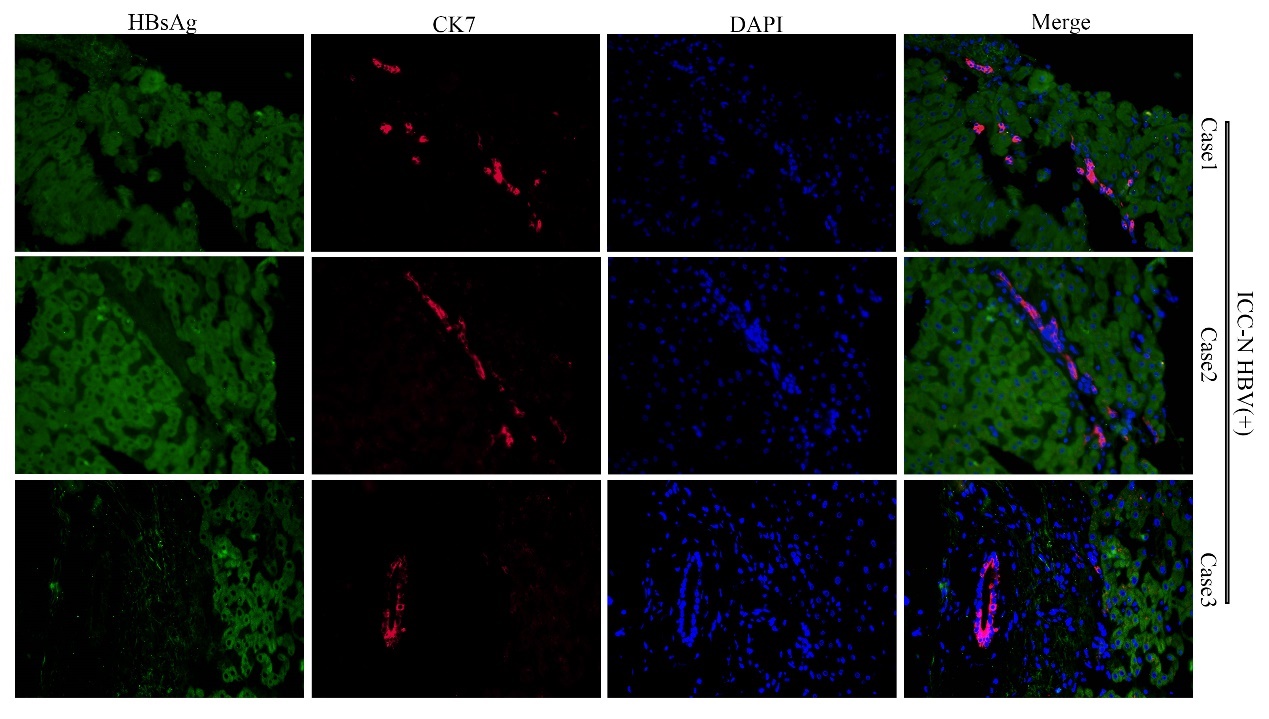


Supplementary figure 6: CK7 was expressed in the bile duct cells in the portal area. HBsAg was expressed in hepatocytes, but not in the bile duct cells.
